# Gut microbiota of ring-tailed lemurs (*Lemur catta*) vary across natural and captive populations and correlate with environmental microbiota

**DOI:** 10.1186/s42523-022-00176-x

**Published:** 2022-04-28

**Authors:** Sally L. Bornbusch, Lydia K. Greene, Sylvia Rahobilalaina, Samantha Calkins, Ryan S. Rothman, Tara A. Clarke, Marni LaFleur, Christine M. Drea

**Affiliations:** 1 Department of Evolutionary Anthropology, Duke University, Durham, NC USA; 2 Duke Lemur Center, Durham, NC USA; 3 Faculty of Sciences, University of Antananarivo, Antananarivo, Madagascar; 4 Department of Psychology, Program in Animal Behavior and Conservation, Hunter College, New York, NY USA; 5 Institute for the Conservation of Tropical Environments, Interdepartmental Doctoral Program in Anthropological Sciences, Stony Brook University, Stony Brook, NY USA; 6 Department of Sociology and Anthropology, North Carolina State University, Raleigh, NC USA; 7 Department of Anthropology, University of San Diego, 5998 Alcala Park, San Diego, CA USA

## Abstract

**Background:**

Inter-population variation in host-associated microbiota reflects differences in the hosts’ environments, but this characterization is typically based on studies comparing few populations. The diversity of natural habitats and captivity conditions occupied by any given host species has not been captured in these comparisons. Moreover, intraspecific variation in gut microbiota, generally attributed to diet, may also stem from differential acquisition of environmental microbes—an understudied mechanism by which host microbiomes are directly shaped by environmental microbes. To more comprehensively characterize gut microbiota in an ecologically flexible host, the ring-tailed lemur (*Lemur catta*; n = 209), while also investigating the role of environmental acquisition, we used 16S rRNA sequencing of lemur gut and soil microbiota sampled from up to 13 settings, eight in the wilderness of Madagascar and five in captivity in Madagascar or the U.S. Based on matched fecal and soil samples, we used microbial source tracking to examine covariation between the two types of consortia.

**Results:**

The diversity of lemur gut microbes varied markedly within and between settings. Microbial diversity was not consistently greater in wild than in captive lemurs, indicating that this metric is not necessarily an indicator of host habitat or environmental condition. Variation in microbial composition was inconsistent both with a single, representative gut community for wild conspecifics and with a universal ‘signal of captivity’ that homogenizes the gut consortia of captive animals. Despite the similar, commercial diets of captive lemurs on both continents, lemur gut microbiomes within Madagascar were compositionally most similar, suggesting that non-dietary factors govern some of the variability. In particular, soil microbial communities varied across geographic locations, with the few samples from different continents being the most distinct, and there was significant and context-specific covariation between gut and soil microbiota.

**Conclusions:**

As one of the broadest, single-species investigations of primate microbiota, our study highlights that gut consortia are sensitive to multiple scales of environmental differences. This finding begs a reevaluation of the simple ‘captive vs. wild’ dichotomy. Beyond the important implications for animal care, health, and conservation, our finding that environmental acquisition may mediate aspects of host-associated consortia further expands the framework for how host-associated and environmental microbes interact across different microbial landscapes.

**Supplementary Information:**

The online version contains supplementary material available at 10.1186/s42523-022-00176-x.

## Introduction

The structure of gut microbial communities within vertebrates is influenced in part by endogenous host factors, such as genotype and physiology [1–3], and in part by exogenous factors, such as sociality, seasonality, habitat quality, and diet [4–6]. These exogenous factors can influence which microbial taxa in a gut community thrive or become depauperate, as amply demonstrated in dietary studies [7–10], or they can provide opportunities for more direct routes of microbial acquisition [11–14]. For example, the transmission of microbes between hosts, as evidenced by horizontal pathogen transfer [15–17] or vertical transmission during the birthing process and nursing [18, 19], are significant drivers of host health and development. There is, likewise, the potential for horizontal acquisition of microbes via exposure to environmental consortia on natural (e.g., soil) and man-made surfaces, plus on food and in water [12, 20–23]; however, this latter route to shaping host-associated communities, hereafter referred to as ‘environmental acquisition,’ remains understudied. Here, we match-sampled ring-tailed lemur (*Lemur catta*) feces with soil from 13 ‘settings’, to (a) characterize variation in host gut microbiota, (b) characterize variation in soil microbiota, and (c) test for any covariation between host and soil communities. Examining environmental microbes alongside host-associated communities is a first step to understanding the role of environmental acquisition in population-level differences between host microbiomes.

Previous studies of intraspecific variation in gut microbiota, often framed using a ‘wild vs. captive’ comparison, have provided valuable descriptions of differences in presumed extremes [24–26]. For example, researchers often report a ‘signal of captivity,’ whereby the gut microbiota of captive hosts differ significantly from those of wild conspecifics, converging on a perturbed or ‘humanized’ composition [25, 27, 28]. Perturbations of this nature are generally attributed to commercial diets that include manufactured chow and cultivated produce [27, 29, 30]; nevertheless, studies of captive populations have been focused on accredited zoos or rescue facilities that may not represent the range of captive conditions or may be confounded by within-species comparisons across continents [26, 29, 31]. Even comparative field studies have been limited in the number of populations per species studied, typically to a few populations that differ on a given metric of interest (e.g. season, health state, habitat type or quality [32–35]). Because hosts experience a wider range of environmental settings than is typically encompassed within wild vs. captive comparisons, a broader comparative approach is necessary to provide a more comprehensive and nuanced understanding of gut microbial variation.

As noted, differential exposure to environmental microbes provides potential for horizontal transmission and environmental acquisition [20, 22, 23, 36–38], with the ingestion of specific microbes being linked to novel digestive functions of the gut microbiota [39–41]. Under certain scenarios, environmental acquisition has been shown to outweigh vertical transmission as the main mode of microbial colonization [42, 43]. Although environmental acquisition may promote heterogeneity within and between hosts [44], its role rarely has been considered a differentiating factor between wild and captive hosts. Husbandry practices and veterinary care, for example, introduce cleaning products and antibiotics to the microbial environment of captive animals [45, 46], further differentiating it from the ‘native’ environment [47], with potentially critical consequences to microbiome structure and function.

Our study species, the ring-tailed lemur, is a semi-terrestrial, omnivorous strepsirrhine primate [48, 49] that occupies various habitats across southern Madagascar [50] and also survives well in captivity [51]. Its ecological flexibility, coupled with existing knowledge about its gut microbiome [26, 52–54], motivates broader comparative study of intraspecific variation that takes environmental acquisition of microbes into consideration. We therefore collected fecal and soil samples originating from lemurs and their environments, respectively, under three broad, environmental conditions: the wilderness condition in Madagascar (W_M_; 8 settings) represents a large portion of the ring-tailed lemur’s natural habitat, whereas two captivity conditions distributed between Madagascar (C_M_; 2 settings) and the U.S. (C_U.S._; 3 settings) represent a wide range of housing conditions on two continents (spanning pet ownership, zoos, and other facilities; Table 1).

**Table 1:**
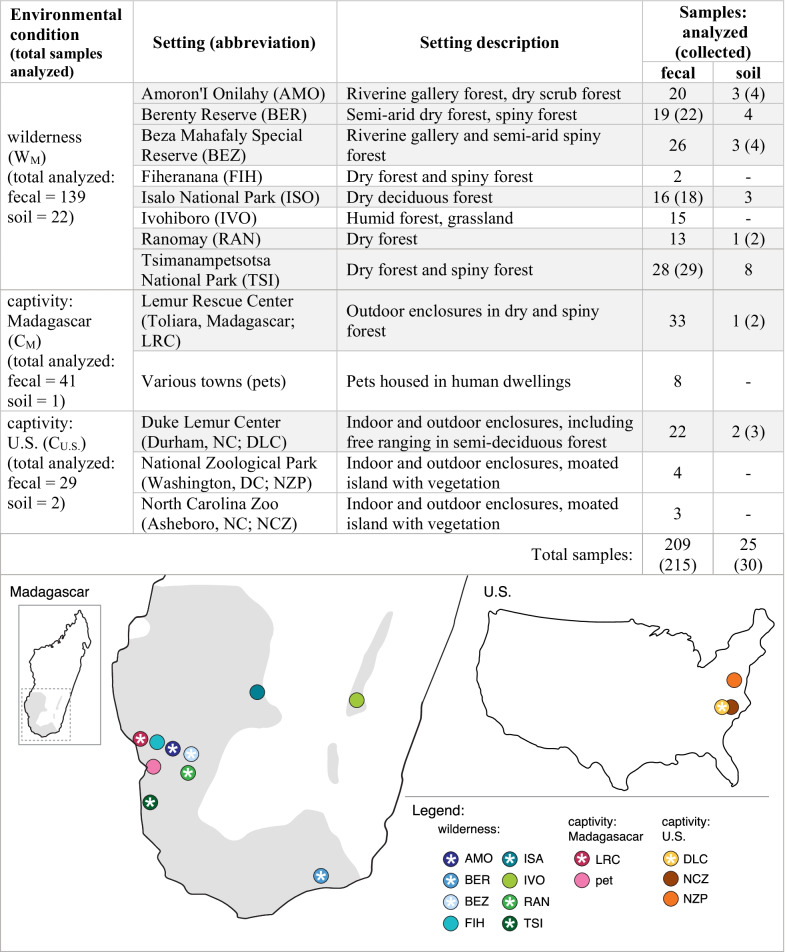
Research settings (names, descriptions, and locations) and samples collected under wilderness conditions and under captivity conditions in Madagascar and the U.S.

A subset of the samples collected were omitted from analyses owing to low-yield extractions or low-quality sequencing. Soil samples could not always be obtained. Settings for which matched fecal and soil samples were analyzed are shaded in gray in the table and have an asterisk in the maps. Maps show locations of each setting; the gray shaded area of the map shows the natural range of wild ring-tailed lemurs in Madagascar

To analyze covariation between lemur gut microbiota and soil microbiota in our 13 settings, we combine 16S rRNA sequencing and statistical tools based on microbial source tracking [55, 56], which is the process of modelling the predicted origin of microbes to a given community (e.g., lemur gut microbiomes) based on certain source communities (e.g., soil samples). Given the variability of environmental factors across our multiple settings, we expect the diversity, membership, and composition of lemur gut microbiota and soil microbiota to differ within and between our three environmental conditions (Table 1).

If diet or habitat quality were the main driver of gut microbiota composition, we would expect (a) wild lemurs to show variation between their natural settings, (b) captive lemurs, regardless of continent, to show similar gut microbiota between their settings (reflecting commercial diets and perturbed habitats), and (c) wild and captive lemurs to differ most drastically from one another, in line with prior studies [27]. If, however, environmental acquisition were to play a major role in shaping lemur gut microbiota, we would again expect (a) wild lemurs to show variation between their natural settings (reflecting the soil microbiota of the lemurs’ habitat), but we would expect (b) Malagasy lemurs (wild and captive) to share certain soil-derived microbiota, differing most drastically from captive lemurs in the U.S., and (c) differential access to soil within captivity conditions to correlate with differential soil-associated microbes present in hosts. With regard to the latter, for example, we might expect greater proportions of soil-associated microbes in captive lemurs that gain access to natural enclosures compared to their counterparts that are housed indoors.

## Results

### Lemur gut microbiota: variation in diversity, membership, and composition

#### Alpha diversity

**Fig. 1:**
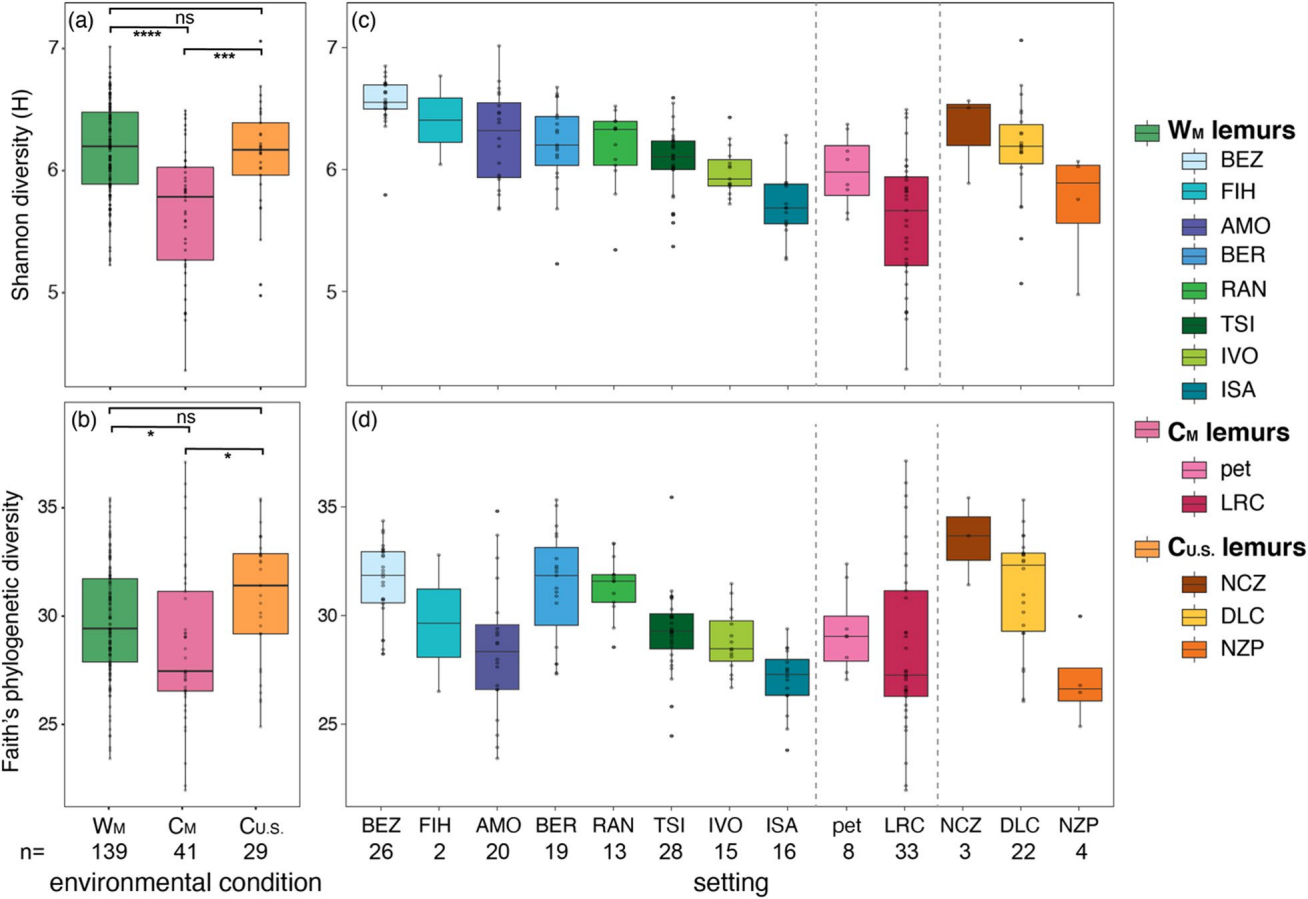
Alpha diversity metrics of gut microbiota (**a**, **b**) collapsed by environmental condition, including from lemurs in the wilderness (W_M_; green), captivity in Madagascar (C_M_; pink), and captivity in the U.S. (C_U.S._; orange), and (**c**, **d**) averaged across individuals for each of the 13 different settings inhabited (reprising the color codes of each condition, delineated by dashed vertical lines). Shown are both (**a**, **c**) Shannon diversity and (**b**, **d**) Faith’s phylogenetic diversity. Across the (**c**, **d**) settings within an environmental condition (see Table 1 for names of abbreviated study settings), the data are plotted in descending order of mean Shannon diversity. Tukey-style box and whiskers show the median (center horizontal line) and the interquartile range (upper and lower bounds of the box), with outliers that are 1.5 times less than the 25th quartile or 1.5 times more than the 75th quartile. Number of samples (n) is reported below each condition and setting. Kruskal–Wallis test with Benjamini–Hochberg correction; *p < 0.05, ***p < 0.001, **** p < 0.0001, ns = nonsignificant. Full statistical results are available in the Additional file 1

Across the gut microbiota of all ring-tailed lemurs sampled in this study, metrics of alpha diversity differed significantly between the three environmental conditions (Generalized Linear Models or GLMs; Shannon: F = 23.773, p < 0.001; Faith’s phylogenetic: F = 4.415, p = 0.013; Fig. 1a, b) and by setting (GLMs; Shannon: F = 13.157, p < 0.001; Faith’s phylogenetic: F = 5.628, p < 0.001; Fig. 1c, d; Additional file 1). The microbiota in fecal samples from W_M_ and C_U.S._ lemurs were similarly diverse overall (pairwise Wilcoxon test; Shannon: p = 0.635; Faith’s phylogenetic: p = 0.056; Fig. 1a, b), whereas those from C_M_ lemurs were significantly less diverse (pairwise Wilcoxon test; Shannon, W_M_ vs. C_M_ lemurs: p < 0.001; W_M_ vs. C_U.S._ lemurs: p < 0.001; Faith’s phylogenetic, W_M_ vs. C_M_ lemurs: p = 0.022; W_M_ vs. C_U.S._ lemurs: p = 0.021; Fig. 1a, b). Within environmental condition, however, both metrics of alpha diversity varied widely between the different settings (Fig. 1c, d; Additional file 1). For example, among W_M_ lemurs, setting was a significant predictor of both metrics of alpha diversity (GLMs; Shannon diversity: F = 20.768, p < 0.001; Faith’s phylogenetic: F = 11.104, p < 0.001). Sex was not a significant predictor in any models of either alpha diversity metric (Additional file 1).

#### Community membership

**Fig. 2:**
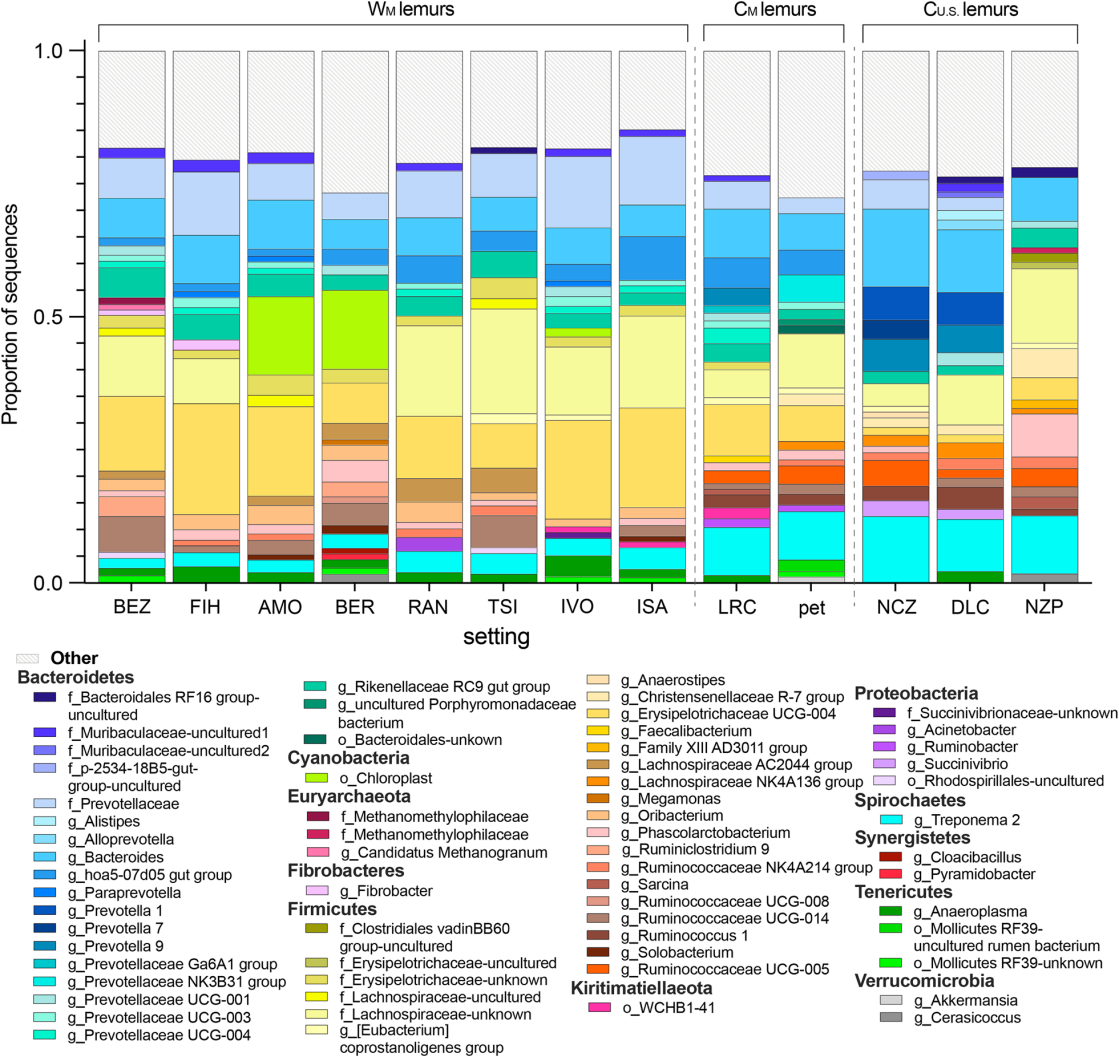
Mean proportion of sequences assigned to microbial taxa across lemurs at each of the 13 different settings, with the three environmental conditions (wilderness, W_M_; captivity in Madagascar, C_M_; and captivity in the U.S., C_U.S._) delineated by dashed vertical lines (see Table 1 for names of abbreviated study settings). Taxa are identified by phylum and deepest possible taxonomic level (i.e., genus level or above); those representing < 1% of the microbiomes were combined into the category “Other”

**Fig. 3:**
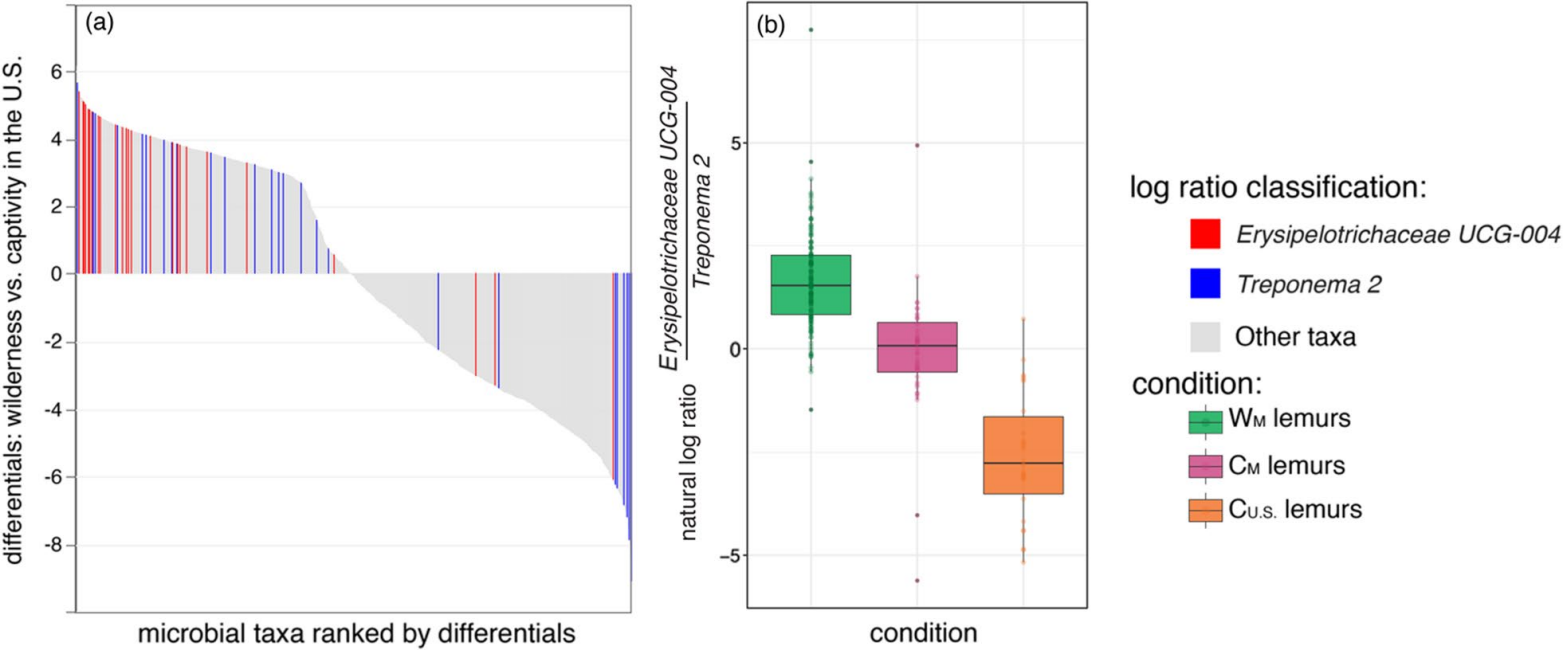
Differential abundance of *Erysipelotrichaceae UCG-004* and *Treponema* 2 amplicon sequence variants (ASVs) in the gut microbiota of lemurs. **a** Differential rank plot showing lemur gut microbial ASVs (x axis) ranked by their differentials (y axis; the estimated log-fold changes for taxa abundances across sample groups) for wild lemurs in Madagascar (W_M_) vs. captive lemurs in the U.S. (C_U.S._). Those ASVs that are more abundant in W_M_ lemurs compared to C_U.S._ lemurs appear on the right side of the plot whereas those that are less abundant in W_M_ lemurs appear on the left side. The differentials of *Erysipelotrichaceae UCG-004* and *Treponema 2* ASVs are highlighted in red and blue, respectively, with other taxa represented in gray. **b** Natural log ratios of *Erysipelotrichaceae UCG-004* versus *Treponema 2* in lemurs across all three environmental conditions. Tukey-style box and whiskers show the median (center horizontal line) and the interquartile range (upper and lower bounds of the box), with outliers that are 1.5 times less than the 25th quartile or 1.5 times more than the 75th quartile. Each point represents a single lemur gut microbiome in which the target ASVs were present

The membership of lemur gut microbiota included 64 abundant taxa (i.e., those that accounted for > 1% of sequences). Of these 64 taxa, only four (6.2%) were shared across lemurs from all settings: the genera *Bacteroides* (phylum Bacteroidetes), *Rikenellaceae RC9 gut group* (Bacteroidetes), *Erysipelotrichaceae* UCG-004 (Firmicutes), and *Treponema 2* (Spirochaetes). Within environmental condition, five (7.8%) taxa were shared by all wild lemurs, whereas 10 (15.6%) and six (9.4%) taxa were shared by C_M_ and C_U.S._ lemurs, respectively (Fig. 2). Using Analysis of Compositions of Microbiomes (ANCOM), we identified 801 amplicon sequence variants (ASVs) that were differentially abundant across the three environmental conditions. For example, members of the Erysipelotrichaceae family characterized the microbiota of W_M_ lemurs, whereas taxa from the Spirochaetaceae and Prevotellaceae families were more abundant in the gut microbiota of captive lemurs from both continents. *Erysipelotrichaceae UCG-004* and *Treponema 2*, for example, were abundant in all lemurs (Fig. 2), but the log ratios of the two genera distinguished lemur gut microbiota by the three environmental conditions and, in particular, differentiated W_M_ lemurs from C_U.S._ lemurs (Fig. 3).

#### Beta diversity

**Fig. 4:**
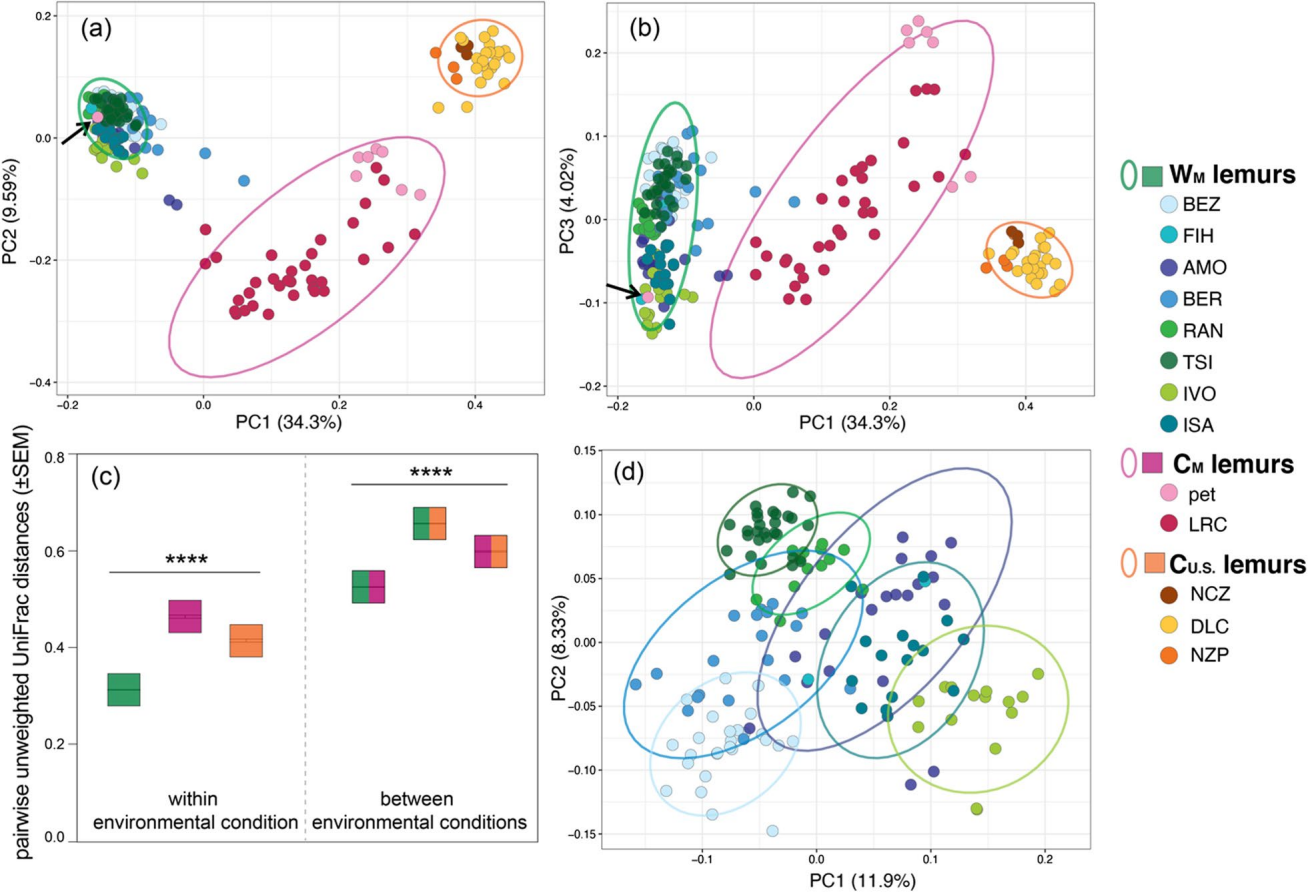
Beta diversity (unweighted UniFrac distances) of lemur gut microbiota across three environmental conditions—wilderness in Madagascar (W_M_; green), captivity in Madagascar (C_M_; pink), and captivity in the U.S. (C_U.S._; orange)—that encompass 13 setting (see Table 1 for names of the abbreviated research settings). **a**, **b** Principal coordinate plots, showing axes 1 and 2, or 1 and 3, respectively, of individual gut microbial communities colored by setting and encircled by normal data ellipses reflecting environmental condition. **c** Mean beta diversity distance scores within an environmental condition (single color) and between two environmental conditions (two colors). The center of the box reflects the mean and the error bars represent ± the standard error of the mean (SEM). **d** Principal coordinate plots, showing axes 1 and 2, for the eight settings within the wilderness condition. Kruskal–Wallis test with Benjamini–Hochberg correction; **** p < 0.0001

The composition of lemur gut microbial communities was significantly distinct across the three environmental conditions, as revealed by beta diversity (Permutational Multivariate Analysis of Variance or PERMANOVA; W_M_ vs. C_M_ lemurs: pseudo-F = 30.169, p < 0.001; W_M_ vs. C_U.S._ lemurs: pseudo-F = 97.912, p < 0.001; C_M_ vs. C_U.S._ lemurs: pseudo-F = 20.808, p < 0.001). Across all subjects, gut microbiota composition clustered distinctly by environmental condition (principal coordinate analysis of unweighted UniFrac distances; Fig. 4a, b). One notable exception, however, owed to a single pet lemur: Unlike its in-country peers (i.e., other C_M_ lemurs), its microbial community structure matched those of W_M_ lemurs (see arrows in Fig. 4a, b).

Across the three environmental conditions, Random Forest Analysis accurately assigned 208 of the 209 gut microbial profiles to the correct environmental condition, with a low (0.48%) out-of-bag (OOB) error rate. Based on its gut microbiota, only the previously mentioned pet lemur (see arrows in Fig.4a, b) was misclassified as a W_M_ lemur. Across the 13 settings, Random Forest Analysis accurately classified 189 of the 209 microbial profiles (OOB error = 9.57%). The gut microbial communities of W_M_ and C_M_ lemurs were misclassified at rates of 7.9% and 7.3%, respectively, whereas those of C_U.S._ lemurs were misclassified at a rate of 20.6%.

With respect to uniformity within environmental condition, the composition of gut microbial communities were least dissimilar between W_M_ lemurs and most dissimilar between C_M_ lemurs (Kruskal–Wallis test; main effect of environmental condition on beta diversity: χ^2^ = 27,487, p < 0.0001; pairwise Wilcoxon test; within W_M_ vs. within C_M_ lemurs: p < 0.001; within W_M_ vs. within C_U.S._ lemurs: p < 0.0001; Fig. 4c). Between environmental conditions, the microbiota of W_M_ and C_M_ lemurs were the least dissimilar, whereas the microbiota of W_M_ vs. C_U.S._ lemurs were the most dissimilar (pairwise Wilcoxon test: ‘W_M_ vs. C_M_’ vs. ‘W_M_ vs. C_U.S._’, p < 0.0001; ‘W_M_ vs. C_M_’ vs. ‘C_M_ vs. C_U.S._’, p < 0.0001; Fig. 4c). Considering W_M_ lemurs only, microbiota composition clustered by setting (Fig. 4d). Although there was some overlap between settings, the patterns are suggestive of microbial ‘signatures’ across different settings.

### Soil microbiota: variation in diversity, membership, and composition

#### Alpha diversity

**Fig. 5:**
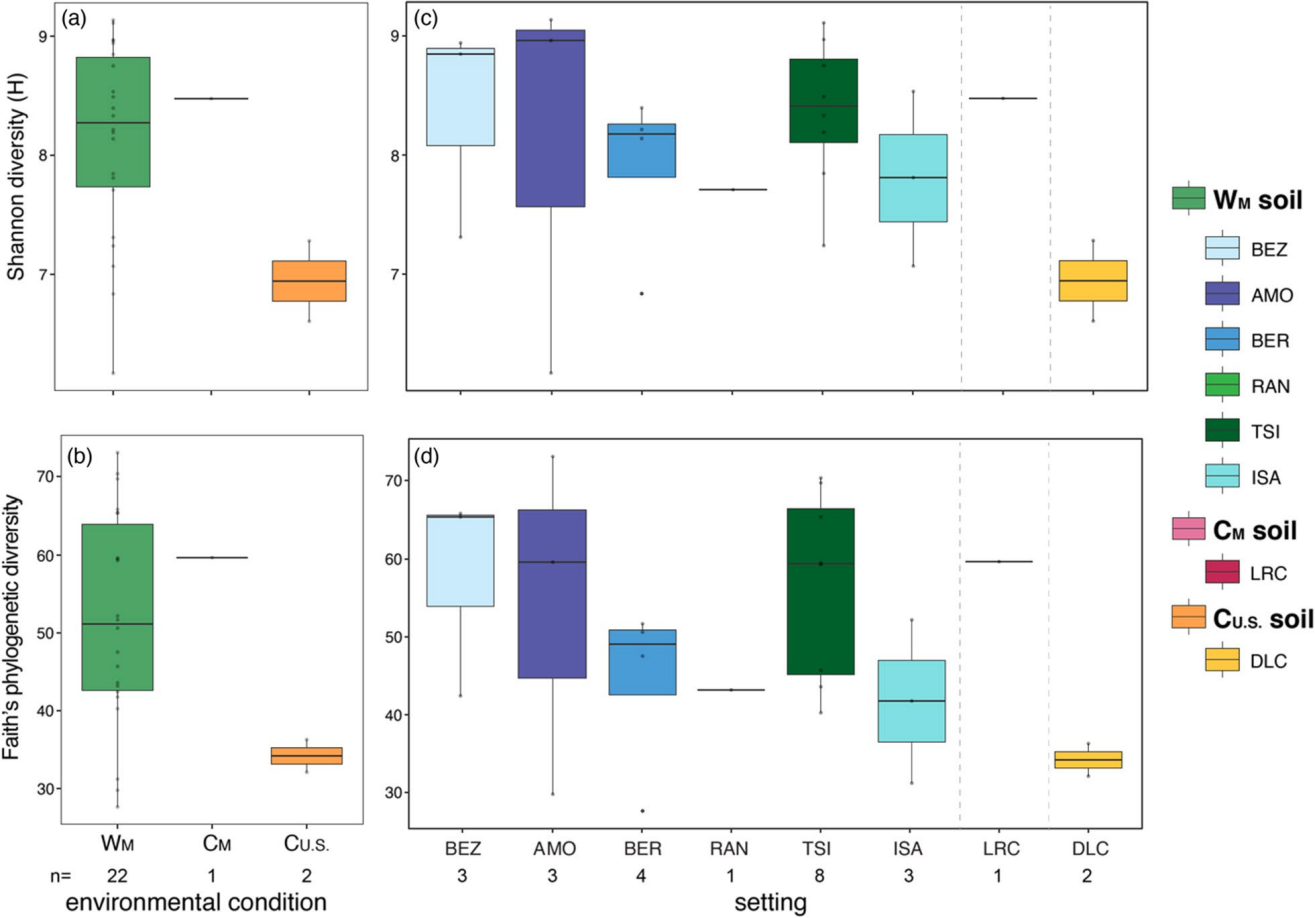
Alpha diversity metrics of soil microbiota (**a**, **b**) collapsed by environmental condition, including the wilderness in Madagascar (W_M_; green), captivity in Madagascar (C_M_; pink), and captivity in the U.S. (C_U.S._; orange) and (**c**, **d**) averaged across individuals for each of the eight different settings (reprising the color codes of each condition, delineated by dashed vertical lines). Shown are both (**a**, **c**) Shannon diversity and (**b**, **d**) Faith’s phylogenetic diversity. Across the (**c**, **d**) settings within a condition (see Table 1 for names of abbreviated research settings), the data are plotted in descending order of mean Shannon diversity. Tukey-style box and whiskers show the median (center horizontal line) and the interquartile range (upper and lower bounds of the box), with outliers that are 1.5 times less than the 25th quartile or 1.5 times more than the 75th quartile. The number of samples (n) is reported below each environmental condition and setting

Across the eight settings for which we sampled soil, the alpha diversity of soil microbiota did not vary significantly between environmental conditions (Kruskal–Wallis test; Shannon diversity: χ^2^ = 3.3457, p = 0.187; Faith’s phylogenetic: χ^2^ = 3.433, p = 0.179; Fig. 5) nor between settings (Kruskal–Wallis test; Shannon diversity: χ^2^ = 7.496, p = 0.379; Faith’s phylogenetic: χ^2^ = 8.936, p = 0.257; Fig. 5). These null findings may owe to small sample sizes.

#### Community membership

**Fig. 6:**
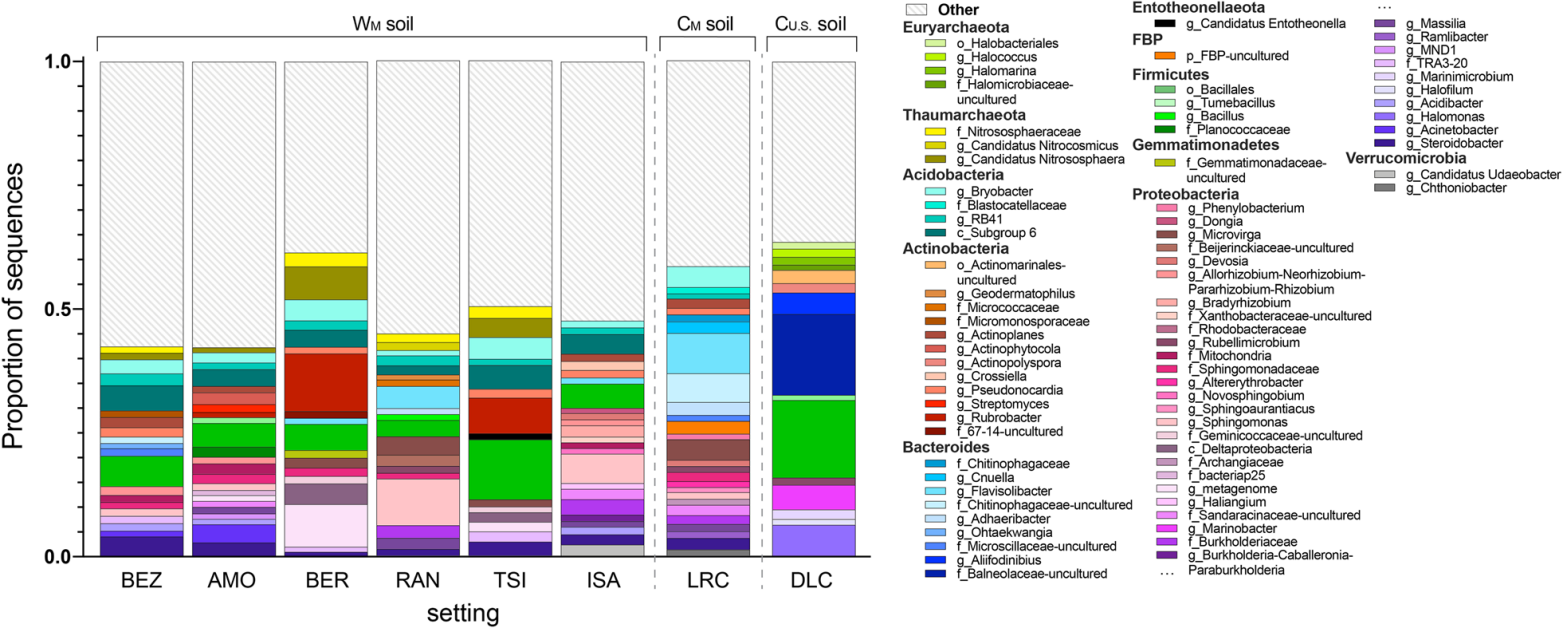
Mean proportion of sequences assigned to microbial taxa of soil at each of the eight settings sampled, within the three environmental conditions: wilderness in Madagascar (W_M_; green), captivity in Madagascar (C_M_; pink), and captivity in the U.S. (C_U.S._; orange), which are delineated by dashed vertical lines (see Table 1 for names of abbreviated research settings). Taxa are identified by phylum and deepest possible taxonomic level (i.e., genus level or above); those representing < 1% of the microbiomes were combined into the category “Other”

The membership of soil communities included 77 abundant taxa, of which none were shared across all settings (Fig. 6). Of the identified soil microbiota, 78.12% were unique to the soil samples and were not found in any lemur fecal samples. For the five wild populations for which we sampled soil, only five abundant taxa were shared: the genera *Bacillus* (phylum Firmicutes), *Steroidobacter* (Proteobacteria), *Bryobacter* (Acidobacteria), and *RB41* (Acidobacteria), and an unidentified member of the class Subgroup 6 (Acidobacteria). ANCOM identified nine ASVs that were differentially abundant across all soil samples, five of which (55.6%) belonged to the Balneolaceae family. In addition, compared to soil from Madagascar (i.e., W_M_ and C_M_), the C_U.S._ soil communities were differentially enriched for the genus *Bacillus*. By contrast, members of the family Nitrososphaeraceae (Thaumarchaeota) and the genus *Acinetobacter* (Proteobacteria) characterized W_M_ soils and C_M_ soils, respectively (Additional file 1).

#### Beta diversity

**Fig. 7:**
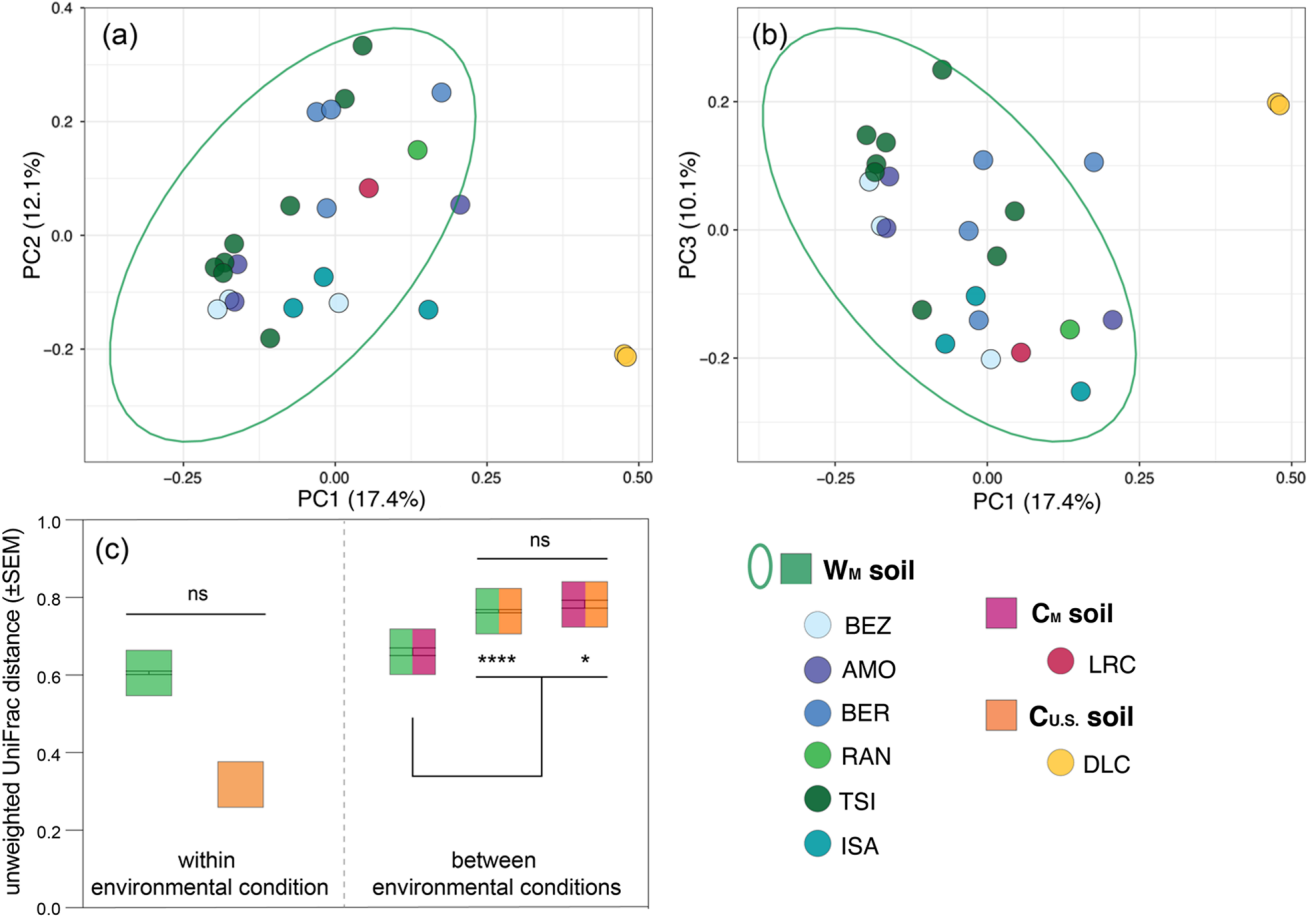
Beta diversity (unweighted UniFrac distances) of soil microbiota across three environmental conditions—wilderness in Madagascar (W_M_; green), captivity in Madagascar (C_M_; pink), and captivity in the U.S. (C_U.S._; orange)—that encompass eight setting (see Table 1 for names of abbreviated research settings). **a**, **b** Principal coordinate plots, showing axes 1 and 2, or 1 and 3, respectively, of soil microbial communities colored by setting and encircled by normal data ellipses reflecting environmental condition. **c** Mean beta diversity distance scores within an environmental conditions (single color) and between two environmental conditions (two colors). The center of the box reflects the mean and the error bars represent ± the standard error of the mean (SEM). Kruskal–Wallis test with Benjamini–Hochberg correction; * p < 0.05, **** p < 0.0001

Despite the small sample sizes, the beta diversity of the soil microbiota varied between environmental conditions (Fig. 7), but only significantly so between W_M_ and C_U.S._ soils (PERMANOVA; W_M_ vs. C_M_ soils: pseudo-F = 1.337, p = 0.202; W_M_ vs. C_U.S_ soils: pseudo-F = 3.897, p = 0.012; C_M_ vs. C_U.S_ soils: pseudo-F = 7.752, p = 0.329). Variation in soil communities within an environmental condition was not significantly different between W_M_ soils or C_U.S._ soils (pairwise Wilcoxon test, p = 0.130; Fig. 7c). Between environmental conditions, W_M_ and C_M_ soils had the lowest dissimilarities (pairwise Wilcoxon test; ‘W_M_ vs. C_M_’ vs. ‘W_M_ vs. C_U.S_’ soils: p < 0.001; ‘W_M_ vs. C_M_’ vs. ‘C_M_ vs. C_U.S_’: p = 0.016; ‘W_M_ vs. C_U.S_’ vs. ‘C_M_ vs. C_U.S_’: p = 0.338 Fig. 7c).

### Covariation of lemur gut and soil microbiota

For analyses of covariation between fecal and soil microbiota, we used samples from the eight settings for which we had matched fecal and soil samples, totaling 177 lemur fecal samples and 25 soils samples (Table 1). There were 191 ASVs shared between lemur fecal communities and soil communities. These were dominated by members of the Firmicutes (75 ASVs or 39.3%), Proteobacteria (49 ASVs or 25.6%), and Bacteroidetes (38 ASVs or 19.9%) phyla. Although many of the shared taxa were abundant (> 1%) in either lemur gut microbiota or soil microbiota, only one genus, *Acinetobacter* (Proteobacteria), was abundant in both lemur gut microbiota and soil microbiota.

**Fig. 8:**
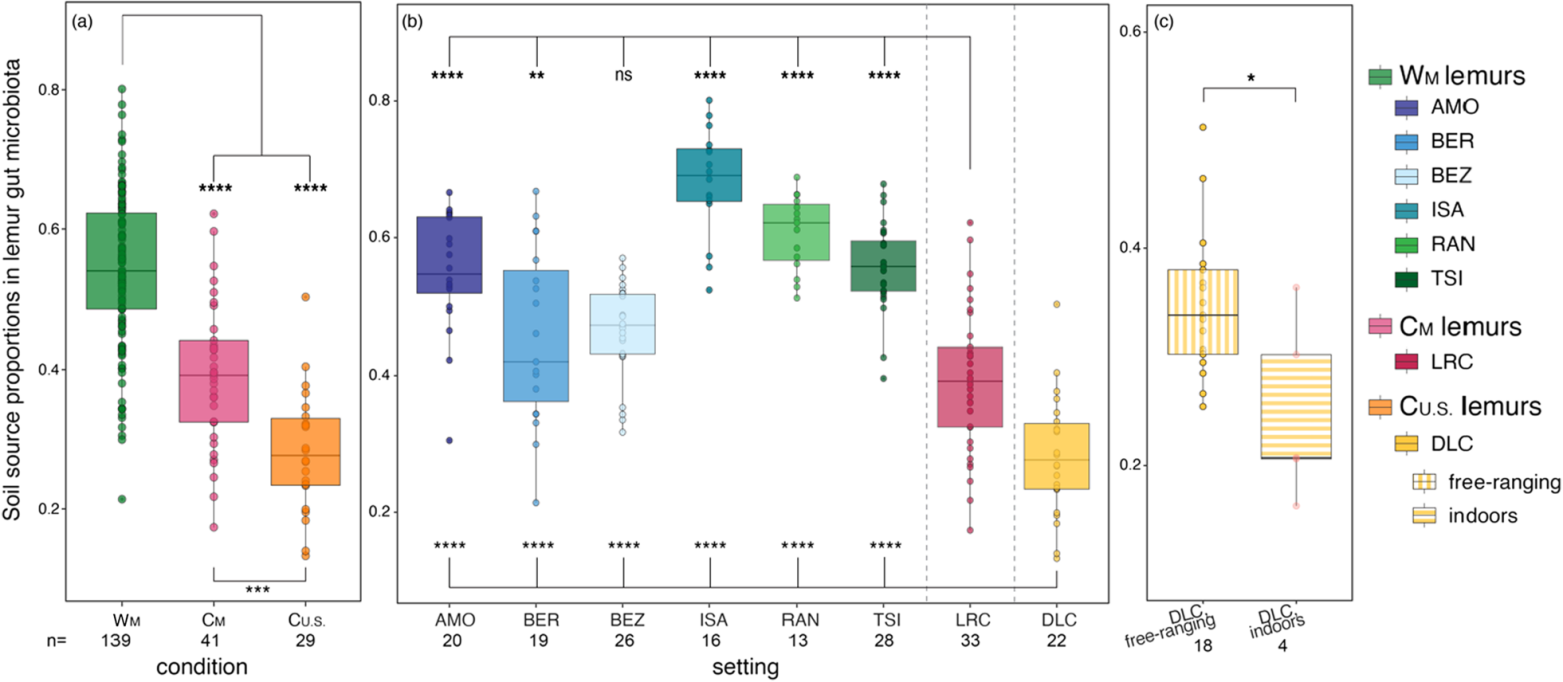
Source proportions, calculated using probabilistic models in FEAST, for soil-associated microbes in the gut microbiota (GMB) of lemurs **a** collapsed by environmental condition: wilderness in Madagascar (W_M_; green), captivity in Madagascar (C_M_; pink), and captivity in the U.S. (C_U.S._; orange), **b** at each of the eight settings for which we had matched fecal and soil samples (reprising the color codes of each condition, delineated by dashed vertical lines), and **c** by housing status (i.e., semi-free-ranging in natural habitat enclosures or housed indoors) at the Duke Lemur Center (DLC). Tukey-style box and whiskers show the median (center horizontal line) and the interquartile range (upper and lower bounds of the box), with outliers that are 1.5 times less than the 25th quartile or 1.5 times more than the 75th quartile. Number of samples (n) is reported below each condition and setting. Kruskal–Wallis test with pairwise comparisons and Benjamini–Hochberg correction; * p < 0.05, ** p < 0.01, *** p < 0.001, **** p < 0.0001, ns = nonsignificant

As would be predicted if environmental acquisition impacts host microbial communities, there was a significant correlation between the abundances of microbes in lemur feces and soil samples (Mantel test; r = 0.494, p < 0.001). The proportion of ‘soil-associated’ microbes found in lemur gut microbiota varied significantly across environmental conditions (Kruskal–Wallis test; χ^2^ = 73.862, p < 0.001; Fig. 8a) and settings (Kruskal–Wallis test; χ^2^ = 112.69, p < 0.001; Fig. 8b). Overall, the gut microbiota of W_M_ lemurs had significantly greater proportions of soil-associated microbes compared to those of all captive lemurs (pairwise Wilcoxon test, p < 0.001; Fig. 8). In addition, C_M_ lemurs had significantly greater proportions of soil-associated microbes in their gut microbiota compared to C_U.S._ lemurs (pairwise Wilcoxon test; p < 0.001; Fig. 8). For lemurs housed at the DLC, those that semi-free-ranged in outdoor, natural habitat enclosures had significantly greater proportions of soil-associated microbes in their gut microbiota compared to lemurs that did not have access to forested enclosures (Kruskal–Wallis test; χ^2^ = 4.641, p = 0.031; Fig. 8c).

**Fig. 9:**
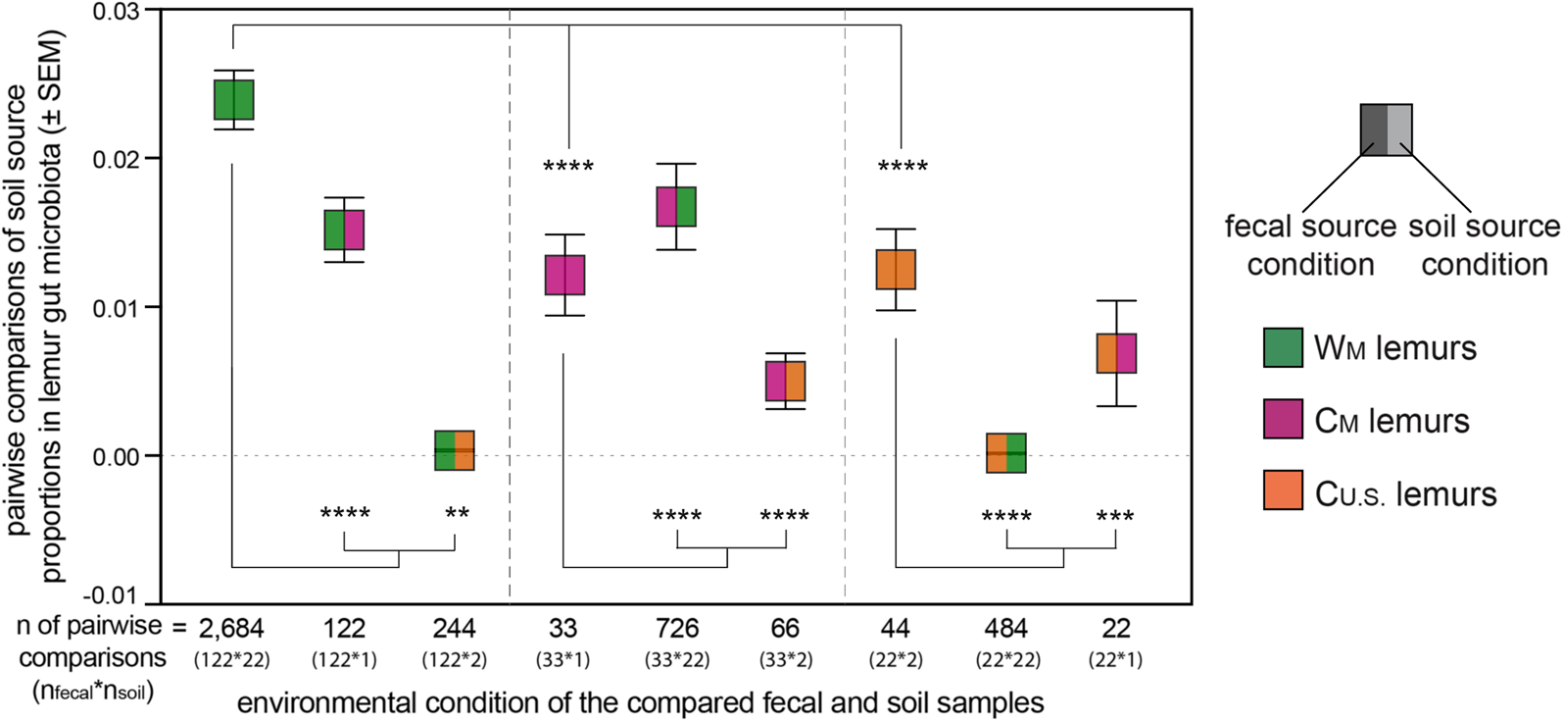
Source proportions, calculated using probabilistic models in FEAST, for soil-associated microbes in the gut microbiota of lemurs within (single color) and between (two colors) the three environmental conditions that encompass eight settings: wilderness in Madagascar (W_M_; green), captivity in Madagascar (C_M_; pink), and captivity in the U.S. (C_U.S._; orange). Within the gut microbiota of lemurs from a given environmental condition (left color = fecal source condition), values show the proportion of soil associated microbes from a given condition (right color = soil source condition). The center of the box reflects the mean and the error bars represent ± the standard error of the mean (SEM). Number of pairwise comparisons and the associated calculation is reported below each box. Kruskal–Wallis test with pairwise comparisons and Benjamini–Hochberg correction; ** p < 0.01, *** p < 0.001, **** p < 0.0001

Soil from within a lemur’s setting accounted for, on average, significantly greater proportions of the lemur’s gut microbiota than did soil communities from other settings (Fig. 9, Additional file 1). Overall, the greatest proportion of soil-associated microbes within lemur gut microbiota occurred when comparing the W_M_ lemurs to W_M_ soils (Fig. 9; Additional file 1). The proportion of soil-associated microbes from C_U.S._ soil that were present in the gut microbiomes of W_M_ lemurs was close to zero (Fig. 9; Additional file 1). Similarly, soil-associated microbes from W_M_ soils were largely absent from the gut microbiome of C_U.S._ lemurs (Fig. 9; Additional file 1). Thus, despite small sample sizes, the greatest differences observed involved the soil microbes from different continents.

## Discussion

Through fecal and soil sampling from multiple settings representing the ring-tailed lemur’s natural range in Madagascar and in captivity on two continents, we have highlighted (1) the wide and often underrepresented variety of gut microbiota present within a single host species, (2) the lack of a universal ‘signal of captivity’ that uniformly decreases microbial diversity, (3) aspects of microbial membership and composition that differ markedly between wild and captive populations, and (4) covariance between lemur gut microbiota and soil microbiota, which points to a key role of environmental microbes. Researchers have reported host ‘group signatures’ in microbiota, often attributed to the social transmission of microbes [5, 57–60]; our results expand this concept to ‘population signatures,’ similar to the widely studied differences across human populations [61, 62], and draw attention to the potential role of environmental acquisition of microbes in mediating significant inter-population variation.

Across populations of W_M_ lemurs, we first observed substantial variation in gut microbial diversity, membership, and composition, indicating that there is not a single ‘representative’ gut community for wild ring-tailed lemurs, as is likely the case for most host species [63]. Nonetheless, the pattern of natural variation observed did not always meet expectations. For example, lemurs living in what is considered a relatively pristine setting, IVO—a recently discovered humid forest patch that is relatively undisturbed by human activity—unexpectedly had the second-lowest diversity of gut microbes. To the extent that lack of disturbance is a proxy of habitat quality, this pattern would be inconsistent with previous reports that greater habitat quality promotes more diverse gut microbiota [64, 65]. In prior studies, the gut microbiota of ring-tailed lemurs were relatively unaffected by habitat degradation [52]. Therefore, either pristine habitats can be of low quality or the ecological and dietary flexibility of this species may dampen the impact of variation in habitat quality and type, relative to more specialized primates (e.g., folivores) [26, 66–68]. That we found significant, natural, inter-population variation in a relatively hardy and robust species [50, 69] suggests that hosts with greater sensitivity to environmental variation, including habitat quality and type, would likely show even greater variation than that described herein. If so, studies constrained to single or few host populations are likely to underrepresent the wide-scale, natural variation in host gut microbiota.

Contrary to many previous studies [70–73], but consistent with others [74–77], we did not observe the gut microbiota of captive lemurs to be consistently less diverse than those of wild lemurs. Such inconsistencies raise questions about the commonly held view that greater alpha diversity is both a hallmark of wild individuals and a proxy for a healthier gut community [78–82]. Although we did not assess gut health, we note that pet lemurs are prone to disease [83–85]. Often housed solitarily indoors, in close contact with people and domestic animals, pet lemurs are fed diets of rice and fruit; yet, their gut consortia were as diverse as those of wild lemurs living at the relatively pristine setting, IVO. Moreover, C_U.S._ lemurs from the DLC and NCZ had diverse gut consortia, on par with that seen in the most diverse W_M_ lemurs (e.g., in BEZ lemurs). These results add to the mounting evidence [66, 86, 87] that alpha diversity alone should not be used to extrapolate the health state of gut consortia or the quality of the host’s environment.

We further found that gut microbiota of captive lemurs were not compositionally homogenized by commercial diets [72, 88]. Heterogeneous gut microbiota could reflect slight differences in the diets provided (as the produce and browse available differ between captivity settings), but such minor dietary variation is unlikely to be the sole driver of such marked microbial differences, particularly in an omnivorous host. Non-dietary factors must have contributed to distinguishing the gut communities of captive lemurs. Indeed, the gut microbiota of C_M_ lemurs were compositionally more similar to those of their wild counterparts than to those of C_U.S._ lemurs Based on this observation, we suggest that the effect of a commercial diet is not necessarily the strongest differentiator of gut consortia and that the effects of captivity cannot be standardized across populations.

Beyond diet, other ‘environmental’ aspects of captivity, including conspecific interactions, contact with humans, habitat exposure, and medications (such as antibiotics) are known to impact animal gut microbiomes [25, 54]. Indeed, in a previous study of healthy ring-tailed lemurs at the DLC, researchers demonstrated the long-term, disruptive influence of antibiotics on the gut microbiome [54]. It is likely that C_U.S._ lemurs experience such disruption more frequently than do C_M_ lemurs, particularly pets, that rarely, if ever, receive antibiotic treatment.

Host genotype is also a well-established mediator of microbial community structure. Reduced genetic diversity, evidenced as founder effects or inbreeding depression, plays a variable role across taxa in shaping the gut microbiome [89–92] and may contribute to differences between captive and wild populations. Although both neutral heterozygosity and genomic functional diversity decrease over time in captive ring-tailed lemurs [93, 94], inbreeding effects can be mitigated through managed breeding programs, resulting in the rapid ‘rescue’ of genetic diversity [94]. Lacking genetic information on all populations, we could not address this influence in the present study. Genetic distance between populations also influences gut microbial structure [91, 95]. We would therefore expect the lemurs in Madagascar, whether wild or captive, to be genetically more similar than either group would be to the C_U.S._ lemurs, as the latter have been genetically isolated from wild populations for many generations. Host genetic distance may contribute to explaining some of the variation observed in microbiome structure.

We also found that, between wild and captive lemurs, the membership and composition of gut microbiota was indicative of the environmental condition. There was little evidence of a diverse ‘core’ microbiome, as only four taxa were found to be abundant across all lemur populations. Two of those core taxa, *Erysipelotrichaceae UCG-004* and *Treponema 2*, were differentially abundant between the three environmental conditions. Despite links between members of *Erysipelotrichaceae* and high-fat, commercial diets in humans [96], *Erysipelotrichaceae* microbes were reported to be enriched in wild compared to captive chimpanzees [97], mirroring our findings in lemurs. Furthermore, the genus *Erysipelotrichaceae UCG-004* was more abundant in the gut microbiota of chimpanzees, relative to humans [98], and in folivorous woolly lemurs compared to other lemur species [99]. The functionally diverse members of the *Treponema* genus were more abundant in the gut microbiota of captive vs. wild hosts in other species [97, 100]. *Treponema* members break down pectin [101, 102], a complex plant polysaccharide enriched in ripe fruits, such as those commonly provided to captive ring-tailed lemurs [103, 104].

Compositionally, the gut microbiota of wild lemurs were markedly less varied than those of lemurs in captivity settings, particularly compared to C_M_ lemurs (i.e., pets and LRC lemurs, most of which are former pets). These findings support the “Anna Karenina” principle [105, 106], which posits that perturbations of microbiota result in unstable communities and, thus, perturbed hosts have greater variation in their microbiota than do unperturbed hosts. Indeed, among our lemur populations, the most clearly perturbed animals were the pets or former pets, given their combined experience of translocation, dietary change, and anthropogenic disturbance, leading to perturbed microbial communities that vary greatly between individuals. A single exception to the gut microbiota clustering according to the hosts’ environmental conditions was a pet lemur with gut microbiota that resembled that of wild lemurs. Although we can only speculate about this individual’s history, if recently taken from its natural habitat, the gut microbiota could still reflect the wild origins of this animal, potentially indicative of gradual change in an omnivore’s response to environmental shifts [107, 108].

Lastly, we observed that patterns in lemur gut microbiota were reflected in the diversity and composition of soil microbiota, suggesting that environmental conditions other than diet, including exposure to external microbes in soils, may influence gut microbiomes [109]. Madagascar’s geographical isolation for ~ 88 million years accounts for high levels of floral and faunal endemism [110, 111]. The same is true of microbes, as evidenced by the numerous, unique pathogenic microorganisms found on the island [112–115]. Undoubtedly, variation in nutrients, mineral content, pH, and other abiotic properties of soil further contribute to differentiating soil microbiota across small and large biogeographical scales [116]. Unsurprisingly, therefore, soil microbiota in Madagascar, whether originating in wilderness or captivity settings, were similar in composition and significantly divergent from soils in the U.S. [117]. Given the disparate geographic distributions of many wild vs. captive animals, environmental acquisition that reflects local microbial endemism may be particularly relevant for distinguishing gut microbiota between wild and captive conspecifics. For example, the natural ranges of most primates occur in the tropics [118, 119], yet most accredited zoos and captive facilities that house primates are found outside of tropical regions (in e.g., Europe and North America) [120, 121]; the distinct environmental consortia surrounding wild and captive conspecifics should reflect their geographic or continental divides.

Regarding the exposure to environmental microbes, soil-associated microbes were more prevalent in lemurs that had greater exposure to natural environments and the acquired soil microbes were specific to the lemurs’ environment, reflecting active environmental acquisition. This observation expands on findings that abiotic soil properties mediate primate gut microbiota [109]. Wild and captive ring-tailed lemurs perform geophagy (i.e., earth-eating), a behavior that is linked to nutrient and microbial supplementation [122, 123] and is a potential vector for the incorporation of environmental microbes [40]. Similarly, dietary items may act as vessels of soil or environmental microbes [41]; dietary variation across wild and captive lemurs may influence gut microbiomes by simultaneously offering different nutrients and different microbes.

Difficulties extracting DNA from soil samples reduced our sample sizes, particularly for the captive settings, such that we likely underestimated the variation in soil microbiota within and between environments. Akin to most cross-sectional studies of microbiomes, we were also unable to assess the persistence or viability of the soil-associated microbes in lemur gut communities. It is, therefore, possible that the soil-associated microbes in lemur guts were ephemeral or non-viable; however, our results indicate setting-specific, environmental acquisition, supporting that these patterns are not random and that the acquired microbes may be subject to filters that enable the incorporation of only specific microbes [20, 124, 125]. Furthermore, we analyzed these data from the perspective that environmental consortia act as sources of microbes for host-associated communities, but we expect consistent, bidirectional transmission of microbes between hosts and their environments. Ultimately, greater sampling resolution in matched soil and host-associated communities is necessary to reinforce our results and better elucidate the role of environmental acquisition.

While expanding our understanding of the factors that shape host-microbe relationships, these results also have significant potential to inform animal care and conservation strategies. Perturbed microbiota are increasingly recognized as culprits of obesity, gastrointestinal distress, and even associated mortality in captive animals [79, 126–128]. Given that lemurs are among the most endangered mammals on the planet [129], maintaining populations of healthy animals in captivity is an important ‘safety net’ that augments *in-vivo* conservation efforts [130, 131]. We suggest that environmental acquisition may be a key component of ‘rewilding’ or ‘bioaugmenting’ captive animal gut microbiota, a process by which gut consortia can be reshaped to better promote host-microbe symbiosis [26, 130, 132]. Identifying what comprises healthy gut microbiota is a complex and ongoing area of research; nonetheless, we show that environmental acquisition is a potential driver of microbial communities and thus should be considered a relevant path to affecting animal health.

## Conclusions

Even in a relatively robust, omnivorous host, gut microbiota are distinct across populations. This variation reflects environmental variability that is underrepresented by a simple wild vs. captive dichotomy. Moreover, concurrent analysis of lemur gut and soil microbiota supports the premise that environmental acquisition contributes to shaping host-associated microbiota; hosts and their associated microbes are components of a larger landscape that includes interactions with environmental microbes. Together, these results expand our understanding of intraspecific host-microbe dynamics under varying environmental conditions and reinforce the value of broad-scale, comparative investigations of microbial variation within a single host species.

## Methods

### Study sites

Our research sites included 13 settings (one per ‘population’; settings were categorized based on a combination of shared environmental factors and geographic location), grouped under the following three environmental conditions: wilderness in Madagascar (W_M_; 8 settings), captivity in Madagascar (C_M_; 2 settings), and captivity in the U.S. (C_U.S._; 3 settings; Table 1). The wilderness settings occurred in protected areas (e.g., national parks, community-managed reserves) that varied in habitat type (Table 1). The captivity settings in Madagascar included the Lemur Rescue Center (LRC; Toliara, Madagascar), where the animals were socially housed, and various townships that were home to individual pets. Although the pet lemurs were not located in the same geographic location, they were categorized as a single population because of the shared, unique experiences of ‘pethood’, including receipt of commercial diets prepared for human consumption, housing in human dwellings, contact with humans and domestic animals, and isolation from conspecifics, all of which differ significantly from the experiences of the wild lemurs or other captive lemur populations. Lastly, the captivity settings in the U.S. included the North Carolina Zoo (NCZ; Asheboro, NC), the Duke Lemur Center (DLC; Durham, NC), and the National Zoological Park (NZP; Washington, DC). These facilities were comparable to one another, all with socially housed lemurs.

### Subjects

Across all research settings, our subjects included 215 adult, ring-tailed lemurs (82 male, 81 female, 52 of unknown sex; Table 1). The wilderness settings were each occupied by multiple lemur troops, ranging in size from 5 to 24 individuals. Excluding the pets, all captive settings included groups of 2–7 lemurs that had access to indoor and outdoor enclosures, and were provided facility-standardized diets (i.e., fresh produce and commercial chow, freely available water). Certain animals at the LRC and the DLC also had access to natural habitat enclosures that, respectively, consisted of dry and spiny forest (LRC) or North American deciduous and pine hardwood forest (DLC). The pets were kept in human dwellings (i.e., houses or hotels) and were fed fruit, rice, and other foods intended for human consumption.

### Sample collection

During a span of four years (2016–2020), we collected ‘matched’ fecal and soil samples from our subjects and study sites, respectively. Within 8 weeks of fecal or soil collection, the samples were transported to the U.S., where they were stored at − 80 °C, until analysis.

For feces, we opportunistically collected fresh samples, upon the lemur’s observed voiding. In Madagascar, collections occurred during the dry season (May–October) and, in the U.S., collections occurred end of summer through fall (August-November). To avoid soil contamination of the fecal sample, we removed the outer layer of each fecal pellet. We then placed the sample in an Omnigene tube that contained a stabilizing buffer that preserved microbial communities at room temperature for 8 weeks (Omnigene.Gut tube, DNAgenotek, Ontario, Canada [133, 134]). All settings were represented by fecal samples from minimally two lemurs (the maximum number of individuals represented was 33).

When collecting soil in nature, we avoided high-defecation areas (e.g., under sleeping trees) while identifying core areas where lemurs most commonly spent time on the ground. Within these core areas, we demarcated a 2–3 m^2^ area and collected soil from each of five evenly spaced locations, using a clean, individually wrapped, sterile plastic spatula. For each area, the five aliquots of topsoil (top 2–3 cm of soil) were pooled in a single Omnigene tube to create a representative soil sample. Because multiple lemur troops inhabited each of the wilderness settings, in some cases with overlapping core areas, we prioritized collecting soil samples from areas of maximal use. In some cases, we were unable to collect soil samples for every troop that provided fecal samples. At the LRC and DLC, we used the same collection methods to collect soil samples from areas in the natural habitat enclosures where lemurs semi-free-ranged. Because it is illegal to own pet lemurs in Madagascar, we minimized owner concern by collecting only fecal samples for this group. Because of other logistical and analytical constraints (see below), only eight of the 13 settings were represented by usable, pooled soil samples.

### Microbial DNA extraction and sequencing

Following the manufacturer’s protocols for the DNeasy Powersoil kit (QIAGAN, Frederick, MD), we extracted bacterial genomic DNA from fecal and soil samples. We quantified DNA using a Fluorometer (broad-spectrum kit, Qubit 4, Thermo Fisher Scientific, Waltham, MA). Aliquots of extracted DNA were sent to Argonne National Laboratory’s Environmental Sequencing facility (Lemont, IL) for library preparation and amplicon sequencing of the 16S rRNA gene. After amplification of the V4 region with region-specific primers and sample-specific 12-base barcodes, samples were pooled and amplicon libraries were cleaned using AMPure XP Beads. Amplicons were then sequenced on a 151 × 151 base pair Illumina MiSeq run [135].

### Bioinformatics and statistics

We processed the raw sequence data using a previously published bioinformatics pipeline generated in QIIME2 [136]. In brief, we used the pipeline to join forward and reverse reads, demultiplex and quality filter the joined reads (DADA2; PHRED scores indicated no quality trimming was needed), remove non-bacterial sequences (Mitochondria), generate a phylogenetic tree, and assign taxonomy based on 99% sequence similarity (SILVA database [137, 138], ver. 138.1) to generate amplicon sequence variants (ASVs). After quality filtering, samples with fewer than 10,000 sequences were removed from downstream analyses, resulting in 209 fecal samples and 25 soil samples with over 11 million combined reads and an average of ~ 50,000 reads per sample. To visually represent rare taxa that had relative abundances < 1% of the total sequences, we combined them into the conglomerate “Other” category (Figs. 1 and 6). Using tables of ASVs, we calculated metrics of alpha diversity (Shannon and Faith’s Phylogenetic diversity metric) and beta diversity (weighted and unweighted UniFrac distances). We report only on unweighted UniFrac (vs. also weighted) as it gives equal consideration to rare and abundant taxa, allows for better visualization of variation in less abundant taxa, and is most appropriate for testing our hypotheses and predictions.

To test for differences in alpha diversity between the gut microbiota of lemurs under the three environmental conditions and in the 13 settings, we first used generalized linear models (GLMs; glm in R, ver, 4.0.2) with environmental condition or setting and sex as fixed effects. To further test for variation in lemur gut microbiota and soil microbiota alpha diversity, we used nonparametric statistics (e.g., Kruskal–Wallis tests, and pairwise Wilcoxon rank sum tests with ﻿Benjamini–Hochberg adjustment) to perform pairwise comparisons between the various environmental conditions and settings. To identify and test for effects of environmental condition or setting on beta diversity (unweighted UniFrac distances) in lemur fecal and soil microbiota, we used principal coordinate analysis (i.e., to visualize clustering of microbiota composition) and Permutational Multivariate Analysis of Variance (PERMANOVA) in QIIME2. We then performed Random Forest Analysis [139], which is a supervised learning technique that uses decision trees to classify data to specific categories and provides an overall model error rate (out of the bag error or OOB error). To identify microbes enriched in specific groups of samples, we used differential abundance analyses via Analysis of Compositions of Microbiomes (ANCOM) and songbird software [140] in QIIME2, paired with visualization through Qurro [141].

For the eight settings where we obtained matched fecal and soil samples (Table 1), we analyzed covariation between lemur gut microbiota and the associated soil communities by performing a Mantel test on microbial abundance matrices of lemur gut and soil microbiota. Because multiple lemur fecal samples were associated with each soil sample, we created comparable matrices for the Mantel test by averaging the microbial abundances across the fecal samples of lemurs directly associated with a given soil sample, resulting in a single, mean lemur gut community associated with each soil community. For this process, we omitted fecal samples from troops not represented by a soil sample or for which troop identity was unknown.

To test if soil-associated microbes were present in lemur gut microbiota, we used FEAST, a tool for fast expectation–maximization microbial source tracking [55]. FEAST assumes each ‘sink’ sample is a convex combination of known and unknown ‘sources’ and uses multinomial distributions and machine-learning classification to model the microbial source tracking [55]. For this analysis, we used the matched lemur gut and soil samples; all soil samples collected in a given setting were used to represent the potential exposure to environmental microbes experienced by all sampled lemurs in that same setting, regardless of troop identity. Because we were testing whether environmental acquisition influences lemur gut microbiota, and because this analysis requires an assumption of directionality (i.e., from a source to a sink), we categorized soil samples as ‘sources’ and lemur fecal samples as ‘sinks’; however, we acknowledge and discuss the potential for bi-directional transmission of microbes between lemurs and soil. The FEAST output provides 'source proportions' that represent the scaled proportion of each sink sample (fecal) that can be attributed to each source sample (soil) based on FEAST’s probabilistic models [55]. For each lemur fecal sample, we calculated the proportions of microbes that were attributed to each soil community and from a default ‘unknown source’ that accounts for microbes not relevant to soil microbiota. Lastly, we used FEAST to test for differences in the source proportions in the gut microbiota of lemurs at the DLC that were either semi-free-ranging or sequestered to indoor enclosures.

## Supplementary Information

**Additional file 1.** Supplementary materials: (1) Statistical results on alpha diversity in lemur gut microbiota, (2) statistical results on covariation between lemur gut and soil microbiota, and (3) **supplementary Figure 1** showing differential abundance of soil microbes.

## Data Availability

Sequencing reads are available in the National Center for Biotechnology Information's Sequence Read Archive (BioProject ID PRJNA821395). Additional datasets generated and/or analyzed during the current study are available from the corresponding author upon reasonable request.
