# Supplementary material for: Gut microbiota of ring-tailed lemurs (Lemur catta) vary across natural and captive populations and correlate with environmental microbiota
Source: Anim Microbiome. 2022 Apr 28;4:29. doi: 10.1186/s42523-022-00176-x (PMC9052671; doi:10.1186/s42523-022-00176-x)
Supplement: Supplementary file 1 — Additional file 1. Supplementary materials: (1) Statistical results on alpha diversity in lemur gut microbiota, (2) statistical results on covariation between lemur gut and soil microbiota, and (3) supplementary Figure 1 showing differential abundance of soil microbes. [file 42523_2022_176_MOESM1_ESM.docx]

**Supplementary Materials**

Gut microbiota of wild and captive ring-tailed lemurs (*Lemur catta*) vary across populations and correlate with environmental microbiota

Sally L. Bornbusch, Lydia K. Greene, Samantha Calkins, Ryan S. Rothman, Tara A. Clarke, Marni LaFleur, Christine M. Drea

***1. Statistical results on alpha diversity in lemur gut microbiota***

The full statistical results for two metrics of alpha diversity (Shannon and Faith’s Phylogenetic diversity) in lemur gut microbiota are shown in Tables S1-S4. For each metric, we (a) used a generalized linear mixed model (GLMM) to determine the predictive value of environmental condition (wilderness in Madagascar, W_M_; captivity in Madagascar, C_M_; and captivity in the U.S., C_U.S._; Table S1 & S3) and setting (See Table 1 in the main text for names and descriptions of the 13 different settings; Table S2 & S4), and (b) Kruskal-Wallis rank sum tests and pairwise comparisons using Wilcoxon rank sum exact tests, with Benjamini-Hochberg adjustment for post-hoc and pairwise statistical comparisons.

|  | | | | | | | | | | |  | | | | | | | | | | | | | |  | | | | | | | | | | | | | |  | | | | | | | | | | |  | | | | | | | | |  | | | | | | |  | | | | | | | | | |  | | |  | | | | | | |  | | | |  | | |  | | | |  | | | |
| --- | --- | --- | --- | --- | --- | --- | --- | --- | --- | --- | --- | --- | --- | --- | --- | --- | --- | --- | --- | --- | --- | --- | --- | --- | --- | --- | --- | --- | --- | --- | --- | --- | --- | --- | --- | --- | --- | --- | --- | --- | --- | --- | --- | --- | --- | --- | --- | --- | --- | --- | --- | --- | --- | --- | --- | --- | --- | --- | --- | --- | --- | --- | --- | --- | --- | --- | --- | --- | --- | --- | --- | --- | --- | --- | --- | --- | --- | --- | --- | --- | --- | --- | --- | --- | --- | --- | --- | --- | --- | --- | --- | --- | --- | --- | --- | --- | --- | --- | --- | --- |
| **Table S1. Shannon diversity, by condition** | | | | | | | | | | | | | | | | | | | | | | | | | | | | | | | | | | | | | | |  | | | | | | | | | | |  | | | | | | | | |  | | | | | | |  | | | | | | | | | |  | | |  |  |  |  |  |  |  |  |  |  |  |  |  |  |  |  |  |  |  |  |  |  |
|  | | | | | | | | | | |  | | | | | | | | | | | | | |  | | | | | | | | | | | | | |  | | | | | | | | | | |  | | | | | | | | |  | | | | | | |  | | | | | | | | | |  | | |  |  |  |  |  |  |  |  |  |  |  |  |  |  |  |  |  |  |  |  |  |  |
| GLMM: Shannon diversity ~ condition + sex | | | | | | | | | | | | | | | | | | | | | | | | | | | | | | | | | | | | | | | | | | | | | | | | | |  | | | | | | | | |  | | | | | | |  | | | | | | | | | |  | | |  |  |  |  |  |  |  |  |  |  |  |  |  |  |  |  |  |  |  |  |  |  |
|  | | | | | | | | | | |  | | | | | | | | | | | | | |  | | | | | | | | | | | | | |  | | | | | | | | | | |  | | | | | | | | |  | | | | | | |  | | | | | | | | | |  | | |  |  |  |  |  |  |  |  |  |  |  |  |  |  |  |  |  |  |  |  |  |  |
|  | | | | | Df | | | | | | Deviance | | | | | | | | | AIC | | | | | | | | | F | | | | | | | | | p-value | | | | | | | | | | | |  | | | | | | | |  | | | | | | | | | | | |  | | | | | | | |  |  |  |  |  |  |  |  |  |  |  |  |  |  |  |  |  |  |  |  |  |  |  |
| <none> | | | | |  | | | | | | 29.243 | | | | | | | | | 192.27 | | | | | | | | |  | | | | | | | | |  | | | | | | | | | | | |  | | | | | | | |  | | | | | | | | | | | |  | | | | | | | |  |  |  |  |  |  |  |  |  |  |  |  |  |  |  |  |  |  |  |  |  |  |  |
| condition | | | | | 2 | | | | | | 38.099 | | | | | | | | | 230.86 | | | | | | | | | 23.773 | | | | | | | | | 9.57E-10 | | | | | | | | | | | |  | | | | | | | |  | | | | | | | | | | | |  | | | | | | | |  |  |  |  |  |  |  |  |  |  |  |  |  |  |  |  |  |  |  |  |  |  |  |
| sex | | | | | 1 | | | | | | 29.304 | | | | | | | | | 190.61 | | | | | | | | | 0.33 | | | | | | | | | 0.566 | | | | | | | | | | | |  | | | | | | | |  | | | | | | | | | | | |  | | | | | | | |  |  |  |  |  |  |  |  |  |  |  |  |  |  |  |  |  |  |  |  |  |  |  |
|  | | | | | | | | | | |  | | | | | | | | | | | | | |  | | | | | | | | | | | | | |  | | | | | | | | | | |  | | | | | | | | |  | | | | | | |  | | | | | | | | | |  | | |  |  |  |  |  |  |  |  |  |  |  |  |  |  |  |  |  |  |  |  |  |  |
| Kruskal-Wallis rank sum test | | | | | | | | | | | | | | | | | | | | | | | | | | | | | | | | | | | | | | |  | | | | | | | | | | |  | | | | | | | | |  | | | | | | |  | | | | | | | | | |  | | |  |  |  |  |  |  |  |  |  |  |  |  |  |  |  |  |  |  |  |  |  |  |
| Kruskal-Wallis chi-squared = 30.694, df = 2, p-value = 2.162e-07 | | | | | | | | | | | | | | | | | | | | | | | | | | | | | | | | | | | | | | | | | | | | | | | | | | | | | | | | | | |  | | | | | | |  | | | | | | | | | |  | | |  |  |  |  |  |  |  |  |  |  |  |  |  |  |  |  |  |  |  |  |  |  |
|  | | | | | | | | | | |  | | | | | | | | | | | | | |  | | | | | | | | | | | | | |  | | | | | | | | | | |  | | | | | | | | |  | | | | | | |  | | | | | | | | | |  | | |  |  |  |  |  |  |  |  |  |  |  |  |  |  |  |  |  |  |  |  |  |  |
| Pairwise comparisons using Wilcoxon rank sum exact test, with Benjamini-Hochberg adjustment: | | | | | | | | | | | | | | | | | | | | | | | | | | | | | | | | | | | | | | | | | | | | | | | | | | | | | | | | | | | | | | | | | | | | | | | | | | | | | | |  |  |  |  |  |  |  |  |  |  |  |  |  |  |  |  |  |  |  |  |  |  |
|  | | | | | | | | | | | captivity: Madagascar | | | | | | | | | | | | | | captivity: US | | | | | | | | | | | | | |  | | | | | | | | | | |  | | | | | | | | |  | | | | | | |  | | | | | | | | | |  | | |  |  |  |  |  |  |  |  |  |  |  |  |  |  |  |  |  |  |  |  |  |  |
| captivity: US | | | | | | | | | | | 0.00023 | | | | | | | | | | | | | | - | | | | | | | | | | | | | |  | | | | | | | | | | |  | | | | | | | | |  | | | | | | |  | | | | | | | | | |  | | |  |  |  |  |  |  |  |  |  |  |  |  |  |  |  |  |  |  |  |  |  |  |
| wilderness | | | | | | | | | | | 1.30E-07 | | | | | | | | | | | | | | 0.635 | | | | | | | | | | | | | |  | | | | | | | | | | |  | | | | | | | | |  | | | | | | |  | | | | | | | | | |  | | |  |  |  |  |  |  |  |  |  |  |  |  |  |  |  |  |  |  |  |  |  |  |
|  | | | | | | | | | | |  | | | | | | | | | | | | | |  | | | | | | | | | | | | | |  | | | | | | | | | | |  | | | | | | | | |  | | | | | | |  | | | | | | | | | |  | | |  |  |  |  |  |  |  |  |  |  |  |  |  |  |  |  |  |  |  |  |  |  |
| **Table S2. Shannon diversity, by setting** | | | | | | | | | | | | | | | | | | | | | | | | | | | | | | | | | | | | | | |  | | | | | | | | | | |  | | | | | | | | |  | | | | | | |  | | | | | | | | | |  | | |  |  |  |  |  |  |  |  |  |  |  |  |  |  |  |  |  |  |  |  |  |  |
|  | | | | | | | | | | |  | | | | | | | | | | | | | |  | | | | | | | | | | | | | |  | | | | | | | | | | |  | | | | | | | | |  | | | | | | |  | | | | | | | | | |  | | |  |  |  |  |  |  |  |  |  |  |  |  |  |  |  |  |  |  |  |  |  |  |
| GLMM: Shannon diversity ~ setting + sex | | | | | | | | | | | | | | | | | | | | | | | | | | | | | | | | | | | | | | |  | | | | | | | | | | |  | | | | | | | | |  | | | | | | |  | | | | | | | | | |  | | |  |  |  |  |  |  |  |  |  |  |  |  |  |  |  |  |  |  |  |  |  |  |
|  | | | | | | | | | | |  | | | | | | | | | | | | | |  | | | | | | | | | | | | | |  | | | | | | | | | | |  | | | | | | | | |  | | | | | | |  | | | | | | | | | |  | | |  |  |  |  |  |  |  |  |  |  |  |  |  |  |  |  |  |  |  |  |  |  |
|  | | | | | | Df | | | | | | | Deviance | | | | | | | | | | | AIC | | | | | | | | F | | | | | | | | | p-value | | | | | | | | | | |  | | | | | | | | | | | | | | | | | | | | | | | |  | | |  |  |  |  |  |  |  |  |  |  |  |  |  |  |  |  |  |  |  |  |  |  |
| <none> | | | | | |  | | | | | | | 21.291 | | | | | | | | | | | 155.18 | | | | | | | |  | | | | | | | | |  | | | | | | | | | | |  | | | | | | | | | | | | | | | | | | | | | | | |  | | |  |  |  |  |  |  |  |  |  |  |  |  |  |  |  |  |  |  |  |  |  |  |
| setting | | | | | | 9 | | | | | | | 38.099 | | | | | | | | | | | 230.86 | | | | | | | | 13.157 | | | | | | | | | 2.35E-15 | | | | | | | | | | |  | | | | | | | | | | | | | | | | | | | | | | | |  | | |  |  |  |  |  |  |  |  |  |  |  |  |  |  |  |  |  |  |  |  |  |  |
| sex | | | | | | 1 | | | | | | | 21.337 | | | | | | | | | | | 153.53 | | | | | | | | 0.325 | | | | | | | | | 0.569 | | | | | | | | | | |  | | | | | | | | | | | | | | | | | | | | | | | |  | | |  |  |  |  |  |  |  |  |  |  |  |  |  |  |  |  |  |  |  |  |  |  |
|  | | | | | | | | | | |  | | | | | | | | | | | | | |  | | | | | | | | | | | | | |  | | | | | | | | | | |  | | | | | | | | |  | | | | | | |  | | | | | | | | | |  | | |  |  |  |  |  |  |  |  |  |  |  |  |  |  |  |  |  |  |  |  |  |  |
| Kruskal-Wallis rank sum test | | | | | | | | | | | | | | | | | | | | | | | | | | | | | | | | | | | | | | |  | | | | | | | | | | |  | | | | | | | | |  | | | | | | |  | | | | | | | | | |  | | |  |  |  |  |  |  |  |  |  |  |  |  |  |  |  |  |  |  |  |  |  |  |
| Kruskal-Wallis chi-squared = 89.371, df = 12, p-value = 6.535e-14 | | | | | | | | | | | | | | | | | | | | | | | | | | | | | | | | | | | | | | | | | | | | | | | | | | | | | | | | | | |  | | | | | | |  | | | | | | | | | |  | | |  |  |  |  |  |  |  |  |  |  |  |  |  |  |  |  |  |  |  |  |  |  |
|  | | | | | | | | | | |  | | | | | | | | | | | | | |  | | | | | | | | | | | | | |  | | | | | | | | | | |  | | | | | | | | |  | | | | | | |  | | | | | | | | | |  | | |  |  |  |  |  |  |  |  |  |  |  |  |  |  |  |  |  |  |  |  |  |  |
| Pairwise comparisons using Wilcoxon rank sum exact test, with Benjamini-Hochberg adjustment: | | | | | | | | | | | | | | | | | | | | | | | | | | | | | | | | | | | | | | | | | | | | | | | | | | | | | | | | | | | | | | | | | | | | | | | | | | | | | | |  |  |  |  |  |  |  |  |  |  |  |  |  |  |  |  |  |  |  |  |  |  |
|  | | | | | | | | | | |  | | | | | | | | | | | | | |  | | | | | | | | | | | | | |  | | | | | | | | | | |  | | | | | | | | |  | | | | | | |  | | | | | | | | | |  | | |  |  |  |  |  |  |  |  |  |  |  |  |  |  |  |  |  |  |  |  |  |  |
|  | AMO | | | | | | | | BER | | | | | | | | BEZ | | | | | DLC | | | | | | | | FIH | | | | | | | ISO | | | | | | | IVO | | | | | LRC | | | | | | | | NZP | | | | | | NCZ | | | | | | pet | | | | | RAN | | | | |  |  |  |  |  |  |  |  |  |  |  |  |  |  |  |  |  |  |  |  |  |  |
| BER | 0.777 | | | | | | | | - | | | | | | | | - | | | | | - | | | | | | | | - | | | | | | | - | | | | | | | - | | | | | - | | | | | | | | - | | | | | | - | | | | | | - | | | | | - | | | | |  |  |  |  |  |  |  |  |  |  |  |  |  |  |  |  |  |  |  |  |  |  |
| BEZ | 0.017 | | | | | | | | 0.000 | | | | | | | | - | | | | | - | | | | | | | | - | | | | | | | - | | | | | | | - | | | | | - | | | | | | | | - | | | | | | - | | | | | | - | | | | | - | | | | |  |  |  |  |  |  |  |  |  |  |  |  |  |  |  |  |  |  |  |  |  |  |
| DLC | 0.688 | | | | | | | | 0.840 | | | | | | | | 0.000 | | | | | - | | | | | | | | - | | | | | | | - | | | | | | | - | | | | | - | | | | | | | | - | | | | | | - | | | | | | - | | | | | - | | | | |  |  |  |  |  |  |  |  |  |  |  |  |  |  |  |  |  |  |  |  |  |  |
| FIH | 0.688 | | | | | | | | 0.707 | | | | | | | | 0.975 | | | | | 0.737 | | | | | | | | - | | | | | | | - | | | | | | | - | | | | | - | | | | | | | | - | | | | | | - | | | | | | - | | | | | - | | | | |  |  |  |  |  |  |  |  |  |  |  |  |  |  |  |  |  |  |  |  |  |  |
| ISO | 0.000 | | | | | | | | 0.001 | | | | | | | | 0.000 | | | | | 0.002 | | | | | | | | 0.120 | | | | | | | - | | | | | | | - | | | | | - | | | | | | | | - | | | | | | - | | | | | | - | | | | | - | | | | |  |  |  |  |  |  |  |  |  |  |  |  |  |  |  |  |  |  |  |  |  |  |
| IVO | 0.100 | | | | | | | | 0.061 | | | | | | | | 0.000 | | | | | 0.110 | | | | | | | | 0.299 | | | | | | | 0.021 | | | | | | | - | | | | | - | | | | | | | | - | | | | | | - | | | | | | - | | | | | - | | | | |  |  |  |  |  |  |  |  |  |  |  |  |  |  |  |  |  |  |  |  |  |  |
| LRC | 0.000 | | | | | | | | 0.000 | | | | | | | | 0.000 | | | | | 0.000 | | | | | | | | 0.108 | | | | | | | 0.647 | | | | | | | 0.022 | | | | | - | | | | | | | | - | | | | | | - | | | | | | - | | | | | - | | | | |  |  |  |  |  |  |  |  |  |  |  |  |  |  |  |  |  |  |  |  |  |  |
| NZP | 0.171 | | | | | | | | 0.100 | | | | | | | | 0.001 | | | | | 0.114 | | | | | | | | 0.408 | | | | | | | 0.707 | | | | | | | 0.552 | | | | | 0.694 | | | | | | | | - | | | | | | - | | | | | | - | | | | | - | | | | |  |  |  |  |  |  |  |  |  |  |  |  |  |  |  |  |  |  |  |  |  |  |
| NCZ | 0.863 | | | | | | | | 0.778 | | | | | | | | 0.463 | | | | | 0.647 | | | | | | | | 0.843 | | | | | | | 0.071 | | | | | | | 0.197 | | | | | 0.061 | | | | | | | | 0.361 | | | | | | - | | | | | | - | | | | | - | | | | |  |  |  |  |  |  |  |  |  |  |  |  |  |  |  |  |  |  |  |  |  |  |
| pet | 0.157 | | | | | | | | 0.173 | | | | | | | | 0.000 | | | | | 0.295 | | | | | | | | 0.547 | | | | | | | 0.171 | | | | | | | 0.975 | | | | | 0.108 | | | | | | | | 0.519 | | | | | | 0.242 | | | | | | - | | | | | - | | | | |  |  |  |  |  |  |  |  |  |  |  |  |  |  |  |  |  |  |  |  |  |  |
| RAN | 0.688 | | | | | | | | 0.934 | | | | | | | | 0.000 | | | | | 0.777 | | | | | | | | 0.688 | | | | | | | 0.002 | | | | | | | 0.098 | | | | | 0.001 | | | | | | | | 0.110 | | | | | | 0.519 | | | | | | 0.286 | | | | | - | | | | |  |  |  |  |  |  |  |  |  |  |  |  |  |  |  |  |  |  |  |  |  |  |
| TSI | 0.197 | | | | | | | | 0.207 | | | | | | | | 0.000 | | | | | 0.361 | | | | | | | | 0.519 | | | | | | | 0.003 | | | | | | | 0.302 | | | | | 0.000 | | | | | | | | 0.202 | | | | | | 0.427 | | | | | | 0.688 | | | | | 0.302 | | | | |  |  |  |  |  |  |  |  |  |  |  |  |  |  |  |  |  |  |  |  |  |  |
|  |  | | | | | | | | |  | | | | | | | |  | | | | | | |  | | | | | |  | | | | | | | |  | | | | | | | |  | | | | | | | | | | | | | | | | | | | | | | | | | | | | | | | |  |  |  |  |  |  |  |  |  |  |  |  |  |  |  |  |  |  |  |  |  |  |
|  | | | | | | | | | | |  | | | | | | | | | | | | | |  | | | | | | | | | | | | | |  | | | | | | | | | | |  | | | | | | | | |  | | | | | | |  | | | | | | | | | |  | | |  |  |  |  |  |  |  |  |  |  |  |  |  |  |  |  |  |  |  |  |  |  |
| **Table S3: Faith's phylogenetic diversity, by condition** | | | | | | | | | | | | | | | | | | | | | | | | | | | | | | | | | | | | | | | | | |  | | | | | | | |  | | | | | | | | |  | | | | | | |  | | | | | | | | | |  | | |  |  |  |  |  |  |  |  |  |  |  |  |  |  |  |  |  |  |  |  |  |  |
|  | | | | | | | | | | |  | | | | | | | | | | | | | |  | | | | | | | | | | | | | |  | | | | | | | | | | |  | | | | | | | | |  | | | | | | |  | | | | | | | | | |  | | |  |  |  |  |  |  |  |  |  |  |  |  |  |  |  |  |  |  |  |  |  |  |
| GLMM: Faith's phylogenetic diversity ~ condition + sex | | | | | | | | | | | | | | | | | | | | | | | | | | | | | | | | | | | | | | | | | | | | | | | | | | | | | | | | | | | | | | | | | |  | | | | | | | | | |  | | |  |  |  |  |  |  |  |  |  |  |  |  |  |  |  |  |  |  |  |  |  |  |
|  | | | | | | | | | | |  | | | | | | | | | | | | | |  | | | | | | | | | | | | | |  | | | | | | | | | | |  | | | | | | | | |  | | | | | | |  | | | | | | | | | |  | | |  |  |  |  |  |  |  |  |  |  |  |  |  |  |  |  |  |  |  |  |  |  |
|  | | | Df | | | | | | | | | | | Deviance | | | | | | | | | AIC | | | | | | | | | | | F | | | | | | | | | p-value | | | | | | | | | |  | | | | | | | | | | |  | | | | | | | | |  | | | | | | | |  |  |  |  |  |  |  |  |  |  |  |  |  |  |  |  |  |  |  |  |
| <none> | | |  | | | | | | | | | | | 1361.5 | | | | | | | | | 810.62 | | | | | | | | | | |  | | | | | | | | |  | | | | | | | | | |  | | | | | | | | | | |  | | | | | | | | |  | | | | | | | |  |  |  |  |  |  |  |  |  |  |  |  |  |  |  |  |  |  |  |  |
| condition | | | 2 | | | | | | | | | | | 1438 | | | | | | | | | 815.43 | | | | | | | | | | | 4.415 | | | | | | | | | 0.013 | | | | | | | | | |  | | | | | | | | | | |  | | | | | | | | |  | | | | | | | |  |  |  |  |  |  |  |  |  |  |  |  |  |  |  |  |  |  |  |  |
| sex | | | 1 | | | | | | | | | | | 1361.6 | | | | | | | | | 808.64 | | | | | | | | | | | 0.018 | | | | | | | | | 0.891 | | | | | | | | | |  | | | | | | | | | | |  | | | | | | | | |  | | | | | | | |  |  |  |  |  |  |  |  |  |  |  |  |  |  |  |  |  |  |  |  |
|  | | | | | | | | | | |  | | | | | | | | | | | | | |  | | | | | | | | | | | | | |  | | | | | | | | | | |  | | | | | | | | |  | | | | | | |  | | | | | | | | | |  | | |  |  |  |  |  |  |  |  |  |  |  |  |  |  |  |  |  |  |  |  |  |  |
| Kruskal-Wallis rank sum test: | | | | | | | | | | | | | | | | | | | | | | | | | | | | | | | | | | | | | | |  | | | | | | | | | | |  | | | | | | | | |  | | | | | | |  | | | | | | | | | |  | | |  |  |  |  |  |  |  |  |  |  |  |  |  |  |  |  |  |  |  |  |  |  |
| Kruskal-Wallis chi-squared = 10.383, df = 2, p-value = 0.005563 | | | | | | | | | | | | | | | | | | | | | | | | | | | | | | | | | | | | | | | | | | | | | | | | | | | | | | | | | | |  | | | | | | |  | | | | | | | | | |  | | |  |  |  |  |  |  |  |  |  |  |  |  |  |  |  |  |  |  |  |  |  |  |
|  | | | | | | | | | | |  | | | | | | | | | | | | | |  | | | | | | | | | | | | | |  | | | | | | | | | | |  | | | | | | | | |  | | | | | | |  | | | | | | | | | |  | | |  |  |  |  |  |  |  |  |  |  |  |  |  |  |  |  |  |  |  |  |  |  |
| Pairwise comparisons using Wilcoxon rank sum exact test, with Benjamini-Hochberg adjustment: | | | | | | | | | | | | | | | | | | | | | | | | | | | | | | | | | | | | | | | | | | | | | | | | | | | | | | | | | | | | | | | | | | | | | | | | | | | | | | |  |  |  |  |  |  |  |  |  |  |  |  |  |  |  |  |  |  |  |  |  |  |
|  | | | | | | | | | | |  | | | | | | | | | | | | | |  | | | | | | | | | | | | | |  | | | | | | | | | | |  | | | | | | | | |  | | | | | | |  | | | | | | | | | |  | | |  |  |  |  |  |  |  |  |  |  |  |  |  |  |  |  |  |  |  |  |  |  |
|  | | | | | | | C_M_ lemurs | | | | | | | | | | | | C_U.S._ lemurs | | | | | | | | | | | | | |  | | | | | | | | | | | | |  | | | | | | | |  | | | | | | | |  | | | | | | | | |  | | | | | | | | |  |  |  |  |  |  |  |  |  |  |  |  |  |  |  |  |  |  |  |  |  |
| C_U.S._ lemurs | | | | | | | 0.021 | | | | | | | | | | | | - | | | | | | | | | | | | | |  | | | | | | | | | | | | |  | | | | | | | |  | | | | | | | |  | | | | | | | | |  | | | | | | | | |  |  |  |  |  |  |  |  |  |  |  |  |  |  |  |  |  |  |  |  |  |
| W_M_ lemurs | | | | | | | 0.022 | | | | | | | | | | | | 0.056 | | | | | | | | | | | | | |  | | | | | | | | | | | | |  | | | | | | | |  | | | | | | | |  | | | | | | | | |  | | | | | | | | |  |  |  |  |  |  |  |  |  |  |  |  |  |  |  |  |  |  |  |  |  |
|  | | | | | | | | | | | |  | | | | | | | | | | | | | | |  | | | | | | | | | | | | |  | | | | | | | | | | |  | | | | | | | | |  | | | | | | | |  | | | | | | | | | | |  |  |  |  |  |  |  |  |  |  |  |  |  |  |  |  |  |  |  |  |  |  |
|  | | | | | | | | | | | |  | | | | | | | | | | | | | | |  | | | | | | | | | | | | |  | | | | | | | | | | |  | | | | | | | | |  | | | | | | | |  | | | | | | | | | | |  |  |  |  |  |  |  |  |  |  |  |  |  |  |  |  |  |  |  |  |  |  |
| **Table S4: Faith's phylogenetic diversity, by setting** | | | | | | | | | | | | | | | | | | | | | | | | | | | | | | | | | | | | | | | |  | | | | | | | | | | |  | | | | | | | | |  | | | | | | | |  | | | | | | | | | | |  |  |  |  |  |  |  |  |  |  |  |  |  |  |  |  |  |  |  |  |  |  |
|  | | | | | | | | | | | |  | | | | | | | | | | | | | | |  | | | | | | | | | | | | |  | | | | | | | | | | |  | | | | | | | | |  | | | | | | | |  | | | | | | | | | | |  |  |  |  |  |  |  |  |  |  |  |  |  |  |  |  |  |  |  |  |  |  |
| GLMM: Faith's phylogenetic diversity ~ setting + sex | | | | | | | | | | | | | | | | | | | | | | | | | | | | | | | | | | | | | | | | | | | | | | | | | | | | | | | | | | | | | | | | | | | |  | | | | | | | | | | |  | | | | |  | | | |  | | | |  | | | |  | | | |  |
|  | | | | | | | | | | | |  | | | | | | | | | | | | | | |  | | | | | | | | | | | | |  | | | | | | | | | | |  | | | | | | | | |  | | | | | | | |  | | | | | | | | | | |  | | | | |  | | | |  | | | |  | | | |  | | | |  |
|  | | | | Df | | | | | | | | | | | Deviance | | | | | | | | | | | AIC | | | | | | | | | F | | | | | | | | | | p-value | | | | | | | | | |  | | | | | | | | | |  | | | | | | | | | |  | | | | | | |  | | |  | | | |  | | | | | |  |  |  |  |  |  |
| <none> | | | | 1075 | | | | | | | | | | | 786.59 | | | | | | | | | | |  | | | | | | | | |  | | | | | | | | | |  | | | | | | | | | |  | | | | | | | | | |  | | | | | | | | | |  | | | | | | |  | | |  | | | |  | | | | | |  |  |  |  |  |  |
| setting | | | | 9 | | | | | | | | | | | 1438 | | | | | | | | | | | 815.43 | | | | | | | | | 5.628 | | | | | | | | | | 1.08E-06 | | | | | | | | | |  | | | | | | | | | |  | | | | | | | | | |  | | | | | | |  | | |  | | | |  | | | | | |  |  |  |  |  |  |
| sex | | | | 1 | | | | | | | | | | | 1075.3 | | | | | | | | | | | 784.63 | | | | | | | | | 0.041 | | | | | | | | | | 0.839 | | | | | | | | | |  | | | | | | | | | |  | | | | | | | | | |  | | | | | | |  | | |  | | | |  | | | | | |  |  |  |  |  |  |
|  | | | | | | | | | | | |  | | | | | | | | | | | | | | |  | | | | | | | | | | | | |  | | | | | | | | | | |  | | | | | | | | |  | | | | | | | |  | | | | | | | | | | |  | | | | |  | | | |  | | | |  | | | |  | | | |  |
| Kruskal-Wallis rank sum test: | | | | | | | | | | | | | | | | | | | | | | | | | | | | | | | | | | | | | | | |  | | | | | | | | | | |  | | | | | | | | |  | | | | | | | |  | | | | | | | | | | |  | | | | |  | | | |  | | | |  | | | |  | | | |  |
| Kruskal-Wallis chi-squared = 64.518, df = 12, p-value = 3.347e-09 | | | | | | | | | | | | | | | | | | | | | | | | | | | | | | | | | | | | | | | | | | | | | | | | | | | | | | | | | | | |  | | | | | | | |  | | | | | | | | | | |  | | | | |  | | | |  | | | |  | | | |  | | | |  |
|  | | | | | | | | | | | |  | | | | | | | | | | | | | | |  | | | | | | | | | | | | |  | | | | | | | | | | |  | | | | | | | | |  | | | | | | | |  | | | | | | | | | | |  | | | | |  | | | |  | | | |  | | | |  | | | |  |
| Pairwise comparisons using Wilcoxon rank sum exact test, with Benjamini-Hochberg adjustment: | | | | | | | | | | | | | | | | | | | | | | | | | | | | | | | | | | | | | | | | | | | | | | | | | | | | | | | | | | | | | | | | | | | | | | | | | | | | | | | | | | |  | | | |  | | | |  | | |  | | | |  |  |  |
|  | | | | | | | | | | | |  | | | | | | | | | | | | | | |  | | | | | | | | | | | | |  | | | | | | | | | | |  | | | | | | | | |  | | | | | | | |  | | | | | | | | | | |  | | | | |  | | | |  | | | |  | | | |  | | | |  |
|  | | AMO | | | | | | BER | | | | | | | | BEZ | | | | | DLC | | | | | | | FIH | | | | | | | | ISO | | | | | | | IVO | | | | | LRC | | | | | | | | NZP | | | | | NCZ | | | | | | pet | | | | | RAN | | | | |  |  |  |  |  |  |  |  |  |  |  |  |  |  |  |  |  |  |  |  |  |  |  |  |
| BER | | 0.019 | | | | | | - | | | | | | | | - | | | | | - | | | | | | | - | | | | | | | | - | | | | | | | - | | | | | - | | | | | | | | - | | | | | - | | | | | | - | | | | | - | | | | |  |  |  |  |  |  |  |  |  |  |  |  |  |  |  |  |  |  |  |  |  |  |  |  |
| BEZ | | 0.003 | | | | | | 0.886 | | | | | | | | - | | | | | - | | | | | | | - | | | | | | | | - | | | | | | | - | | | | | - | | | | | | | | - | | | | | - | | | | | | - | | | | | - | | | | |  |  |  |  |  |  |  |  |  |  |  |  |  |  |  |  |  |  |  |  |  |  |  |  |
| DLC | | 0.019 | | | | | | 1.000 | | | | | | | | 0.886 | | | | | - | | | | | | | - | | | | | | | | - | | | | | | | - | | | | | - | | | | | | | | - | | | | | - | | | | | | - | | | | | - | | | | |  |  |  |  |  |  |  |  |  |  |  |  |  |  |  |  |  |  |  |  |  |  |  |  |
| FIH | | 0.913 | | | | | | 0.767 | | | | | | | | 0.705 | | | | | 0.751 | | | | | | | - | | | | | | | | - | | | | | | | - | | | | | - | | | | | | | | - | | | | | - | | | | | | - | | | | | - | | | | |  |  |  |  |  |  |  |  |  |  |  |  |  |  |  |  |  |  |  |  |  |  |  |  |
| ISO | | 0.210 | | | | | | 0.000 | | | | | | | | 0.000 | | | | | 0.000 | | | | | | | 0.714 | | | | | | | | - | | | | | | | - | | | | | - | | | | | | | | - | | | | | - | | | | | | - | | | | | - | | | | |  |  |  |  |  |  |  |  |  |  |  |  |  |  |  |  |  |  |  |  |  |  |  |  |
| IVO | | 0.705 | | | | | | 0.015 | | | | | | | | 0.000 | | | | | 0.018 | | | | | | | 1.000 | | | | | | | | 0.015 | | | | | | | - | | | | | - | | | | | | | | - | | | | | - | | | | | | - | | | | | - | | | | |  |  |  |  |  |  |  |  |  |  |  |  |  |  |  |  |  |  |  |  |  |  |  |  |
| LRC | | 0.851 | | | | | | 0.015 | | | | | | | | 0.005 | | | | | 0.018 | | | | | | | 0.886 | | | | | | | | 0.680 | | | | | | | 0.412 | | | | | - | | | | | | | | - | | | | | - | | | | | | - | | | | | - | | | | |  |  |  |  |  |  |  |  |  |  |  |  |  |  |  |  |  |  |  |  |  |  |  |  |
| NZP | | 0.633 | | | | | | 0.016 | | | | | | | | 0.009 | | | | | 0.041 | | | | | | | 0.705 | | | | | | | | 0.886 | | | | | | | 0.190 | | | | | 0.705 | | | | | | | | - | | | | | - | | | | | | - | | | | | - | | | | |  |  |  |  |  |  |  |  |  |  |  |  |  |  |  |  |  |  |  |  |  |  |  |  |
| NCZ | | 0.061 | | | | | | 0.310 | | | | | | | | 0.251 | | | | | 0.251 | | | | | | | 0.600 | | | | | | | | 0.012 | | | | | | | 0.018 | | | | | 0.106 | | | | | | | | 0.120 | | | | | - | | | | | | - | | | | | - | | | | |  |  |  |  |  |  |  |  |  |  |  |  |  |  |  |  |  |  |  |  |  |  |  |  |
| pet | | 0.633 | | | | | | 0.169 | | | | | | | | 0.031 | | | | | 0.080 | | | | | | | 1.000 | | | | | | | | 0.029 | | | | | | | 0.861 | | | | | 0.395 | | | | | | | | 0.203 | | | | | 0.106 | | | | | | - | | | | | - | | | | |  |  |  |  |  |  |  |  |  |  |  |  |  |  |  |  |  |  |  |  |  |  |  |  |
| RAN | | 0.014 | | | | | | 0.886 | | | | | | | | 0.847 | | | | | 0.886 | | | | | | | 0.886 | | | | | | | | 0.000 | | | | | | | 0.001 | | | | | 0.018 | | | | | | | | 0.015 | | | | | 0.251 | | | | | | 0.060 | | | | | - | | | | |  |  |  |  |  |  |  |  |  |  |  |  |  |  |  |  |  |  |  |  |  |  |  |  |
| TSI | | 0.251 | | | | | | 0.018 | | | | | | | | 0.001 | | | | | 0.022 | | | | | | | 1.000 | | | | | | | | 0.000 | | | | | | | 0.406 | | | | | 0.179 | | | | | | | | 0.148 | | | | | 0.015 | | | | | | 0.847 | | | | | 0.002 | | | | |  |  |  |  |  |  |  |  |  |  |  |  |  |  |  |  |  |  |  |  |  |  |  |  |

***2. Statistical results on covariation between lemur gut and soil microbiota***

Table S4. Full statistical results (Wilcoxon rank sum exact tests, with Benjamini-Hochberg adjustment) for comparing mean proportion of soil-associated microbes in the gut microbiota of lemurs within and between the three environmental conditions (wilderness in Madagascar, captivity in Madagascar, and captivity in the U.S.; Figure 9 in the main text).

|  | C_M_ vs. C_M_ | C_M_ vs. C_U.S._ | C_M_ vs. W_M_ | C_U.S._ vs. C_M_ | C_U.S._ vs. C_U.S._ | C_U.S._ vs. W_M_ | W_M_ vs. C_M_ | W_M_ vs. C_U.S._ |
| --- | --- | --- | --- | --- | --- | --- | --- | --- |
| C_M_ vs. C_U.S._ | 8.90E-08 | - | - | - | - | - | - | - |
| C_M_ vs. W_M_ | 2.00E-16 | 6.60E-13 | - | - | - | - | - | - |
| C_U.S._ vs. C_M_ | 0.0367 | 0.00018 | 4.30E-11 | - | - | - | - | - |
| C_U.S._ vs. C_U.S._ | 2.20E-16 | 9.30E-05 | 0.136 | 1.60E-12 | - | - | - | - |
| C_U.S._ vs. W_M_ | 2.00E-16 | 2.00E-16 | 1.30E-05 | 4.80E-12 | 0.001 | - | - | - |
| W_M_ vs. C_M_ | 0.8311 | 4.60E-15 | 2.00E-16 | 0.004 | 2.00E-16 | 2.00E-16 | - | - |
| W_M_ vs. C_U.S._ | 2.00E-16 | 3.30E-10 | 0.084 | 3.30E-11 | 0.064 | 0.403 | 2.00E-16 | - |
| W_M_ vs. W_M_ | 3.50E-15 | 3.50E-15 | 2.00E-16 | 4.60E-10 | 0.00051 | 0.00011 | 2.00E-16 | 0.007 |

***3. Supplementary Figure 1: Differential abundance of soil microbes***


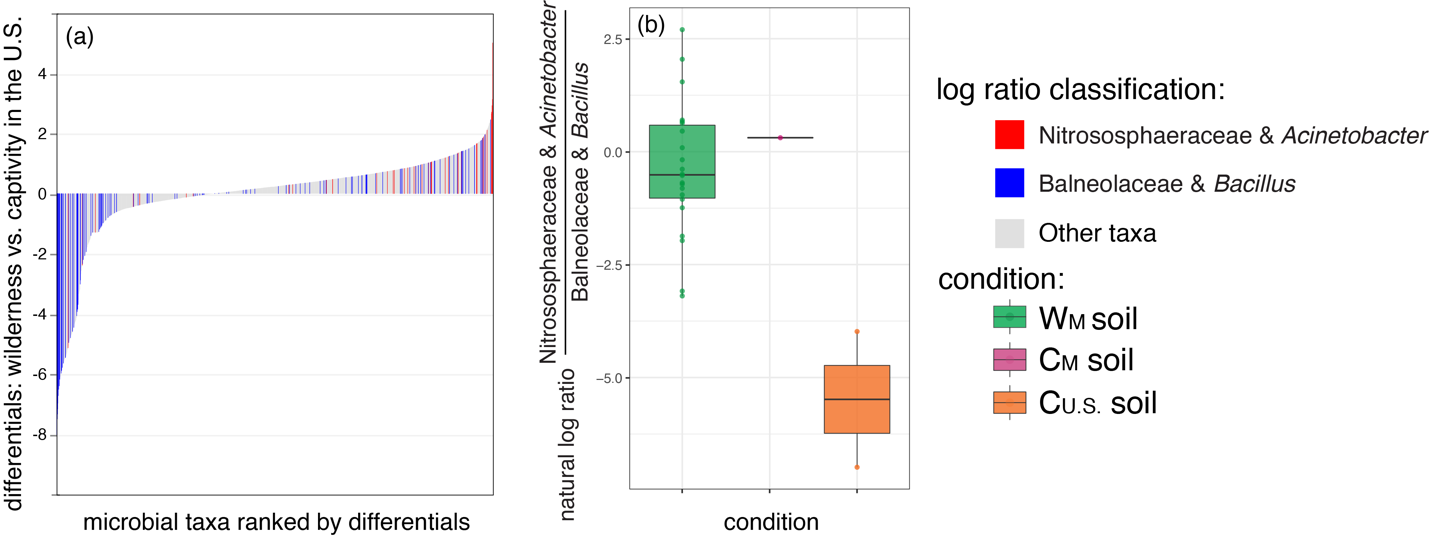


Figure S1. Differential abundance analysis of amplicon sequence variants (ASVs) in the family Nitrososphaeraceae and genus *Acinetorbacter* vs. the family Balneolaceae and genus *Bacillus* in soil microbiota. (a) Differential rank plot showing all microbial ASVs (x-axis) ranked by their differentials (y-axis; the estimated log-fold changes for taxa abundances across sample groups) for soil in the wilderness of Madagascar (W_M_ ) vs. in captivity in the U.S. (C_U.S._). Those ASVs that are more abundant in the wild lemurs compared to captive lemurs in the U.S. appear on the right side of the plot whereas those that are less abundant in wild lemurs appear on the left side. The differentials of ASVs belonging to family Nitrososphaeraceae and genus *Acinetorbacter* are highlighted in red whereas those in the family Balneolaceae and genus *Bacillus* are in blue (other soil taxa are in gray). (b) Natural log ratios of relative abundances of the two groups of taxa across soil from the three environmental conditions. Tukey-style box and whiskers show the median (center horizontal line) and the interquartile range (upper and lower bounds of the box), with outliers that are 1.5 times less than the 25^th^ quartile or 1.5 times more than the 75^th^ quartile. Each point represents a single lemur gut microbiome in which the target ASVs were present.
